# Long-read sequencing reveals genomic diversity and associated plasmid movement of carbapenemase-producing bacteria in a UK hospital over 6 years

**DOI:** 10.1099/mgen.0.001048

**Published:** 2023-07-05

**Authors:** Leah W. Roberts, David A. Enoch, Fahad Khokhar, Grace A. Blackwell, Hayley Wilson, Ben Warne, Theodore Gouliouris, Zamin Iqbal, M. Estée Török

**Affiliations:** ^1^​ European Molecular Biology Laboratory's European Bioinformatics Institute (EMBL-EBI), Wellcome Genome Campus, Hinxton, UK; ^2^​ Department of Medicine, University of Cambridge, England, UK; ^3^​ Clinical Microbiology & Public Health Laboratory, UK Health Security Agency, Cambridge, UK; ^4^​ Centre for Therapeutic Immunology and Infectious Diseases, University of Cambridge, Cambridge, UK; ^5^​ Sanger Institute, Wellcome Genome Campus, Hinxton, UK; ^6^​ Department of Infectious Diseases, Cambridge University Hospitals NHS Foundation Trust, Cambridge, UK

**Keywords:** carbapenemase, ST78, long-read sequencing, plasmid, transmission, whole genome sequencing

## Abstract

Healthcare-associated infections (HCAIs) affect the most vulnerable people in society and are increasingly difficult to treat in the face of mounting antimicrobial resistance (AMR). Routine surveillance represents an effective way of understanding the circulation and burden of bacterial resistance and transmission in hospital settings. Here, we used whole-genome sequencing (WGS) to retrospectively analyse carbapenemase-producing Gram-negative bacteria from a single hospital in the UK over 6 years (*n*=165). We found that the vast majority of isolates were either hospital-onset (HAI) or HCAI. Most carbapenemase-producing organisms were carriage isolates, with 71 % isolated from screening (rectal) swabs. Using WGS, we identified 15 species, the most common being *Escherichia coli* and *Klebsiella pneumoniae *. Only one significant clonal outbreak occurred during the study period and involved a sequence type (ST)78 *K *. *pneumoniae* carrying *bla*_NDM-1_ on an IncFIB/IncHI1B plasmid. Contextualization with public data revealed little evidence of this ST outside of the study hospital, warranting ongoing surveillance. Carbapenemase genes were found on plasmids in 86 % of isolates, the most common types being *bla*_NDM_- and *bla*_OXA_-type alleles. Using long-read sequencing, we determined that approximately 30 % of isolates with carbapenemase genes on plasmids had acquired them via horizontal transmission. Overall, a national framework to collate more contextual genomic data, particularly for plasmids and resistant bacteria in the community, is needed to better understand how carbapenemase genes are transmitted in the UK.

## Data Summary

The datasets [Illumina fastq reads, Oxford Nanopore basecalled (fastq) reads and hybrid assemblies] for this article are available in the European Nucleotide Archive (ENA) under Project PRJEB31034 at https://www.ebi.ac.uk/ena/browser/view/PRJEB30134. The software packages used to analyse these data are described in the Methods section of the paper. The clinical metadata, sample IDs and sample accessions are described in Tables S1 and S2, available in the online version of this article.

**Impact Statement**

Infections caused by bacteria that are resistant to last-line antibiotics (such as carbapenems) can spread easily among patients, are more difficult to treat and are often associated with higher mortality than infections caused by antibiotic-sensitive bacteria. Early detection of these carbapenem-resistant bacteria is therefore essential in healthcare settings. We conducted a study of all carbapenem-resistant bacterial samples in a UK teaching hospital over a 6 year period. We found that most of the carbapenem-resistant samples came from hospital inpatients, or were healthcare-associated, and were detected by screening (using rectal swabs). We used DNA sequencing to identify the bacterial species, antibiotic resistance genes and plasmids (pieces of DNA carrying antibiotic resistance genes). We identified one outbreak caused by a highly antibiotic-resistant strain (* Klebsiella pneumoniae* sequence type 78, which carried the *bla*_NDM-1_ gene on an IncFIB/IncHI1B plasmid). We also found carbapenemase genes on plasmids in 86 % of samples, and evidence of transmission between different bacteria (horizontal transmission). These findings emphasize the potential threat of these organisms and the need for enhanced surveillance for these organisms in healthcare settings.

## Background

Antimicrobial resistance (AMR) is a well-recognised global threat, exacerbated by the overuse and misuse of antibiotics and poor infection control practices [1]. While resistance in bacteria can manifest in different ways, gene-based resistance is of particular concern, as horizontal gene transfer via mobilizable elements can mediate rapid spread of resistance in a population [2, 3].

Carbapenems are a class of broad-spectrum antimicrobial agents that are used to treat a range of infections. Over the last three decades, resistance to carbapenems has spread worldwide via an array of carbapenemase genes [4]. The consequence of this proliferation has been problematic in healthcare settings, with outbreaks of carbapenemase-producing bacteria reported globally [5–7].

Prior to 2000, the incidence of carbapenem-non-susceptible *Enterobacterales* in the UK was low [8]. Between 2003 and 2015, the main carbapenemase genes in circulation in the UK were *bla*_KPC_ followed by *bla*_OXA-48_ and *bla*_NDM_. Following 2015, the number of reported carbapenemase-producers grew substantially, with the proportion shifting slightly in favour of *bla*_OXA-48_, with reduced numbers of *bla*_KPC_ [9]. *Klebsiella* spp. and *Escherichia coli* remain the two species most often associated with carbapenemase genes [10]. To date, whole genome sequencing (WGS) has been used successfully in the UK to elucidate local outbreaks or to investigate targeted resistance mechanisms [11–15]. However, most of these studies have focused on a single carbapenemase gene and/or species [16, 17], and few have explored the complete range of carbapenemase genes and species from a single location over a significant period of time. This is important information at both an institutional and a national level, as it can provide valuable epidemiological evidence for the prevalence and circulation of carbapenemase genes locally, and subsequently be combined with existing public data to explore more widespread transmission.

In this study, we collected all carbapenemase-producing Gram-negative bacteria isolated in the diagnostic microbiology laboratory at Cambridge University Hospitals NHS Foundation Trust (CUH) between 2014 and 2020. Using both Illumina and Oxford Nanopore sequencing, we aimed to characterize the breadth of carbapenemase genes and species encountered in this setting and elucidate the exact genetic context of the carbapenemase genes and possible transmission of strains and/or plasmids. With this knowledge, we can improve our understanding of endemic carbapenemase genes, track their movements and design better infection control strategies.

## Methods

### Study design, setting and participants

This was a prospective observational cohort study conducted at CUH, a 1000 bed secondary and tertiary referral hospital in the UK. Clinical diagnostic samples and screening (rectal swab or stool) samples from hospital inpatients as well as samples referred from local General Practices (GPs), were collected from 1 November 2014 to 31 December 2020. In most cases, a single isolate for each unique species per patient was included in this study. Carbapenemase-producing *Enterobacterales* (CPE) screening polices within CUH changed during the course of the study. In 2014, CPE screening was based on risk factors as defined in the Public Health England guidance [18]. Full screening details can be found in File S1 (Supplementary Methods). Bacterial species were identified using MALDI-TOF MS (Bruker Diagnostics). Isolates with reduced susceptibility to carbapenems were detected either through routine antimicrobial susceptibility testing of clinical diagnostic samples or the use of selective media (details in File S1: Supplementary Methods). Onset definitions are described in File S1 (Supplementary Methods).

### Bacterial culture, DNA extraction and sequencing

Carbapenemase-producing isolates were retrieved from the diagnostic microbiology laboratory archive and cultured on selective media (CHROMID CARBA SMART; bioMérieux). Antimicrobial susceptibility profiles were determined using the VITEK-2 system (bioMérieux). Genomic DNA was extracted using the QICube and the QIAamp 96 DNA QiACube HT kit (QIAgen). In total, 85/165 isolates were available for sequencing with both short (Illumina HiSeq) and long [Oxford Nanopore Technologies (ONT) MinION] read technologies (details in File S1: Supplementary Methods).

### Sequencing quality control and assembly

Illumina raw reads were checked for quality and contamination using fastQC (v0.11.8) [19], MultiQC (v1.9) [20] and Kraken2 (v2.0.9-beta) [21]. Illumina raw reads were filtered using fastp (v0.20.1) [‘--average_qual 30 --length_required 80’] [22]. All Nanopore fast5 reads were basecalled and demultiplexed with Guppy (v3.6.0), using the ‘high-accuracy’ basecalling model and the relevant barcoding kit for demultiplexing. Basecalled reads were then filtered for quality using Nanofilt (v2.7.1) [23] at a threshold of Q7 and a minimum read length of 1000 bp. Isolates with >110× coverage were additionally subsampled down to 100× coverage using Filtlong (v0.2.0) (with --keep_percent 90 and --target_bases corresponding to 100× coverage for that isolate) [24]. All isolate Nanopore reads (original and subsampled) were *de novo* assembled using Flye (v2.8) (with --plasmids flag) [25], Unicycler (v0.4.8) (hybrid settings) [26] and Canu (v2.1.1) (default) [27]. The best assembly per isolate was selected, with a preference for Flye>Unicycler>Canu when the assemblies were comparable. Full details are provided in File S1 (Supplementary Methods).

### Multilocus sequence type (MLST), resistance genes and plasmid profiles

MLST was determined using the hybrid assemblies and the tool mlst (v2.19.0) [28] with the following profiles: (* Acinetobacter baumannii* Pasteur*, Citrobacter freundii, Enterobacter, Escherichia coli* Achtman*, Klebsiella pneumoniae, Pseudomonas aeruginosa*) [29]. Resistance genes and plasmid profiles were detected using the complete assemblies and Abricate (v1.0.1) [30] against the CARD (date: 27 March 2021) [31] and Plasmidfinder (date: 24 october 2020) [32] databases using a minimum coverage of 90 % and minimum nucleotide identity of 90 %. Tandem duplication of carbapenemase genes was confirmed by comparing the duplicated region to the raw (fastq) nanopore reads using BLASTn (v2.12.0) [33].

### Species identification

In addition to Kraken2 (v2.0.9-beta), we also evaluated species identification for *Klebsiella* isolates using Kleborate (v0.4.0-beta) [34, 35] against the complete assemblies at default settings. *Enterobacter* isolates were also manually checked against reference *Enterobacter* species (NCBI) by comparing chromosomes using fastANI (v1.3) [36] with default settings.

### Clonal cluster analysis

Clonal clusters were confirmed using three methods: Snippy (v4.6.0) [37] against a single reference for each species, Split Kmer Analysis (SKA v1.0.1) [38] and MASH (v.2.2.2) [39]. For Snippy, trimmed Illumina reads were mapped to a single reference chromosome for each species (the NCBI reference genome). Clusters at the species level with a low number of pairwise SNPs (<50) were re-tested with Snippy using an internal reference (to obtain a more accurate SNP distance). Complete chromosomes were also clustered using (i) MASH (sketch size 10 000) and (ii) SKA (default settings). Based on existing literature [40–42] and pairwise distances observed in our dataset (File S1: Fig. S1), the following thresholds were used: <20 SNPs (Snippy), MASH threshold 0.001, <25 SNPs and nucleotide identity of 95 (SKA). The final clusters were derived using a combination of the SKA and Snippy results.

### Plasmid clustering

Complete plasmid sequences were clustered using Mash (v2.2.2) using a sketch size of 10 000 and a clustering threshold of 0.001 and 0.005 mash distance. Additional metadata, including plasmid incompatibility type [determined using PlasmidFinder (date: 24 October 2020) and Abricate v1.0.1] and plasmid length, were used to determine the likelihood of the two plasmids being ancestrally related within a short timeframe.

### *K. pneumoniae* phylogeny

In total, 350 publicly available closely related isolates were downloaded from the European Nucleotide Archive (ENA). These were identified by sketching a reference genome (cpe058) using sourmash (v3.5.0) [43] at a Kmer length of 31 bp and an interval of 5000 bp (‘-n 5000 k 31’) and querying against the sourmash index of 661 405 bacterial genomes [44] (full details in File S1: Supplementary Methods). All isolates were trimmed using fastp (v0.20.1) using the following settings: ‘--length_required 40 --cut_front --cut_tail -M 25’. After trimming, 12 isolates were removed for being <20× or >300× coverage. The 338 remaining were combined with trimmed reads for the 11 ST78 outbreak isolates from this study and simulated reads for an Indian ST78 isolate (NCBI: NZ_JAJBIZ010000100.1) [45] as well as three ST78 isolates from Pathogenwatch (accessed 3 May 2023, USA, 2017: SRR13085630 and SRR13085629; Germany, 2019: ERR3771764). Simulated reads were generated using Art_Illumina with the following parameters: ‘--noALN --seqSys HS25 --len 150 --fcov 50 --mflen 500 --sdev 25’ [45]. All reads were mapped to the chromosome of cpe058 and SNPs were called using Snippy (v4.6.0). The core full alignment generated from Snippy-core was then run through Gubbins (v3.3.0) [46] to remove recombination and generate a phylogeny using FastTree (v2.1.10) [47] under the GTRGAMMA model. The final tree was drawn in iTol [48].

## Results

A total of 165 carbapenemase-producing Gram-negative bacterial isolates from 133 patients were collected in the study period. The majority of patients were male (*n*=78, 59 %) (Table 1) and the mean age for both men and women was approximately 54 years. The median length of inpatient stay was 18 days (range 0–156 days) and the majority of patients were alive on discharge (*n*=94, 71 %). Nine isolates (from nine patients) were collected from GPs in the Cambridgeshire area. The remainder were collected from CUH inpatients.

**Table 1.** Baseline characteristics of study participants

Patients (*n*=133)	Male (*n*=78)	Female (*n*=55)
**Age (years)**		
Average	53.7	54.5
Median	57	55
Range	0–95	2–92
Length of stay (days)*		
Min.	0	
Median	18	
Average	26.6	
Max.	156	
**Classification†**		
Community-associated	7	
Hospital-onset	54	
Healthcare-associated	69	
CUH	23	
CUH and overseas hospital	3	
Overseas hospital	17	
Other UK hospital	24	
Other UK and overseas hospital	2	
Unknown:	3	
**History of travel (previous 3 months)**	Travel only	Travel and hospitalization
Asia	3	18
Africa	1	5
Continental Europe	2	9
Middle East	0	1
UK	3	0
Not recorded (*n*=7)		
**Hospitalization (previous 12 months)**	Single site only	Multiple hospitalization sites
CUH	51	7 (overseas)
Other UK hospital	37	3 (overseas)
Overseas hospital	23	0
Not recorded (*n*=1)		
**Outcome (at hospital discharge)**		
Alive	94	
Deceased	13	
Not recorded	9	
Not applicable‡	17	

*Not applicable (GP sample): 9 patients; not applicable (no admission): 8 patients; unknown: 9 patients.

†Definitions: community-associated: isolated <48 h after admission – no previous hospitalization or medical treatment in past 12 months; hospital onset: isolated ≥48 h after admission; healthcare associated: isolated <48 h after admission and had previous hospitalization or medical treatment in last 12 months at (i) CUH, (ii) other UK hospital, (iii) overseas (last 3 months), or a combination of any of the three.

‡Patient was not admitted, so no discharge outcome.

The majority of patients were classified as having either hospital-onset (*n*=54, 41 %) or healthcare-associated (*n*=69, 52 %) infections (Table 1). Of these, roughly a third of patients had exposure to meropenem in the previous 3 months (19/54 hospital-onset, 22/69 healthcare-associated). Only seven (5 %) were community-associated (two from screening swabs, five from urine samples). In the 3 months prior to admission, 39 patients reported travel overseas (29 %) and three to other parts of the UK (2 %). Overall, most patients had been hospitalized within the last year, either in CUH (*n*=51, 38 %), in other UK hospitals (*n*=37, 28 %), or overseas (*n*=23, 17 %). Ten patients had both UK and overseas hospitalizations (seven in CUH and three in other UK hospitals).

### High frequency of carbapenemase gene carriage

**Fig. 1.**
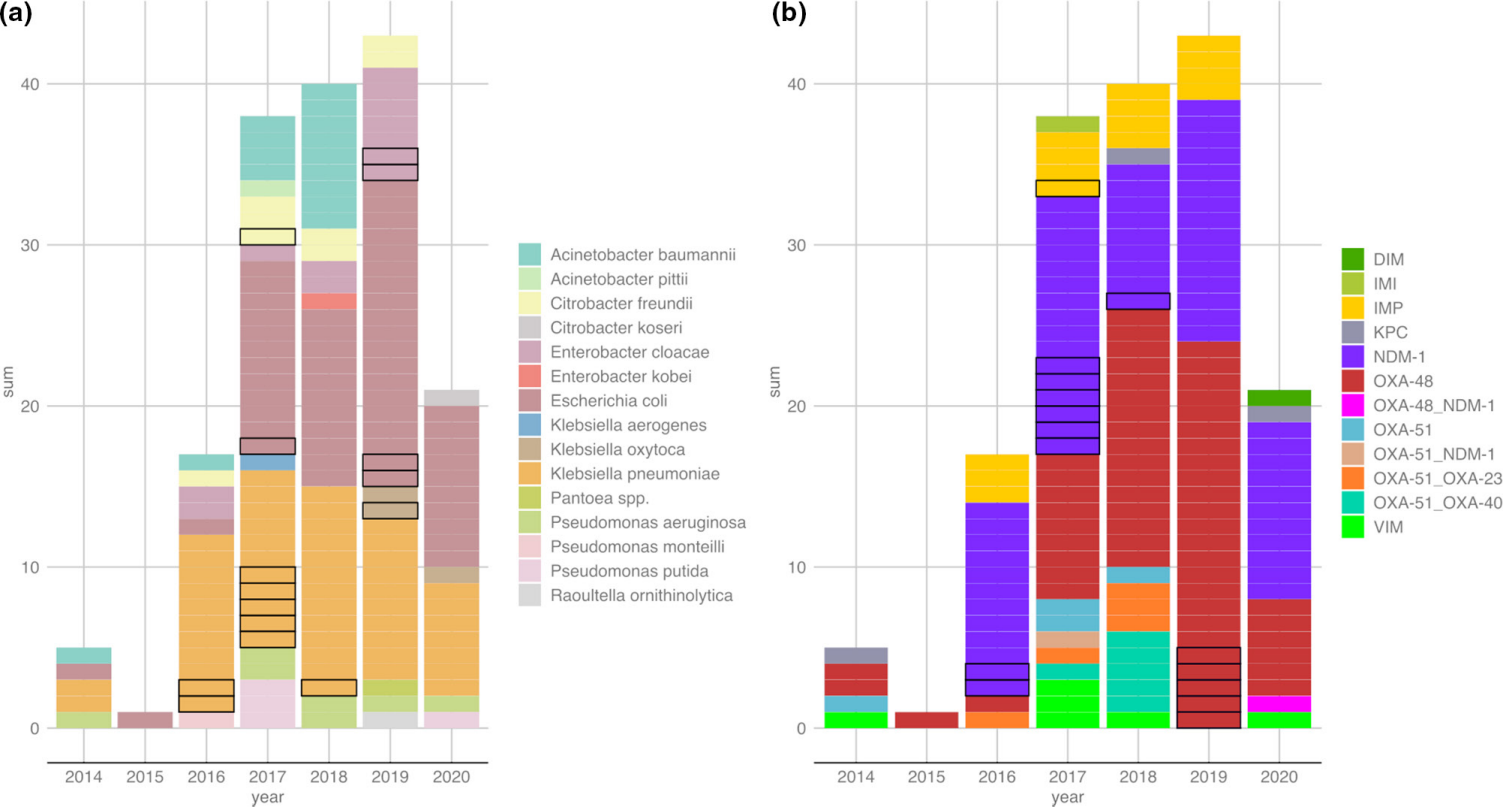
All carbapenemase-positive isolates collected over the study period and carbapenemase genes (*n*=165). Stacked bar plots showing (a) (left) count of species collected per year (determined using MALDI-TOF MS) and (b) (right) count of carbapenemase genes identified per year (determined using PCR). Sequential isolates of the same species (from the same patient) are outlined in black as possible duplicates. Positions of the stacked bars in (a) and (b) are independent.

A total of 15 species were identified during the study, the majority of which were *Klebsiella pneumoniae* (*n*=54, 33 %) or *Escherichia coli* (*n*=55, 33 %) (Fig. 1a). Five patients had recurrent isolates that were included in the study (resulting in the following duplicates: eight *K. pneumoniae *, three *Escherichia coli *, two *Enterobacter* sp*.*, one *Citrobacter freundii* and one *Klebsiella oxytoca *).

The main carbapenemase genes detected were *bla*_NDM-1_ (*n*=65, 39 %) and *bla*_OXA-48_ (*n*=60, 36 %) (Fig. 1b). Most isolates (*n*=117, 71 %) were recovered from screening swabs. The remainder were isolated from urine (*n*=17), blood (*n*=9), biliary fluid (*n*=8), respiratory samples (*n*=6), wound swabs (*n*=5), body fluids (*n*=3), tissue (*n*=2) and central venous catheter tip (*n*=1). There was no association of species or carbapenemase gene with sample site (File S1: Fig. S2). There was also little association between infection classification and species or carbapenemase gene (File S1: Fig. S3). *bla*_NDM-1_- and *bla*_OXA-48_-harbouring *E. coli* and *K. pneumoniae* predominated in all infection categories.

Most isolates were found to have intermediate susceptibility or non-susceptibility to meropenem (File S1: Fig. S4). Notably, *bla*_OXA-48_-carrying isolates had the most variable levels of resistance, with a large portion, in particular *E. coli *, remaining susceptible to meropenem.

### No significant change in the frequency of carbapenemase-producers during the study period

Despite an observed increase per year in carbapenemase-producers (Fig. 1), evaluating the number of isolates obtained per month in the context of adapted sampling procedures over different periods revealed little fluctuation in isolate numbers over time (File S1: Figs S5 and S6). In June 2016 a prospective screening study commenced in the adult intensive care unit (ICU) at CUH and identified an outbreak of *bla*_NDM-1_-positive *K. pneumoniae* (discussed below). This resulted in intensified screening (on admission and weekly thereafter) of all patients admitted to adult ICUs in CUH. A very slight decrease in the number of CPE isolates was observed from late 2019 throughout 2020, probably as a result of the SARS-CoV-2 pandemic (File S1: Fig. S6).

### Travel had little impact on the species isolated or carbapenemase genes detected

When comparing isolates from patients with and without overseas travel, we found a preference for *bla*_NDM_ (particularly *bla*_NDM-1_ *E. coli *) in patients who travelled to Asia, and similarly *bla*_OXA-48_ in patients from central Europe (File S1: Fig. S7). Beyond this, there were very few differences in species and carbapenemase genes isolated based on travel. The most common species/carbapenemase gene combinations (*bla*_NDM-1_- and *bla*_OXA-48_-harbouring *E. coli* and *K. pneumoniae *) were common in patients irrespective of travel history. There was a slightly larger proportion of *bla*_NDM-1_-harbouring *K. pneumoniae* in patients with no travel history; these were related to a hospital outbreak (discussed below).

### Long-read sequencing to generate complete genomes

Of the 165 isolates, 85 (from 68 patients) underwent WGS with both short-read Illumina and long-read nanopore platforms, from which we were able to obtain complete bacterial genomes in most cases (File S2).

Using these genomic data, we re-evaluated the species identifications using Kraken2 as well as Kleborate (for the *Klebsiella* sp.) and fastANI (for *Enterobacter* sp.). All isolates were correct at the genus level, with 12 differences at the species level. These include two reclassifications of *Klebsiella pneumoniae* to *Klebsiella quasipneumoniae* subsp. *similipneumoniae* (cpe097) and *Klebsiella oxytoca* (cpe088), one *Klebsiella oxytoca* to *Klebsiella grimontii* (cpe102), one *Enterobacter kobei* to *Enterobacter hormaechei* (cpe050), five *Enterobacter cloacae* to *Enterobacter hormaechei* (cpe002, icp157, cpe042, cpe090, cpe093), one *Enterobacter cloacae* to *Enterobacter mori* (cpe018), and two *Enterobacter cloacae* to *Enterobacter roggenkampii* (cpe104, cpe106).

**Fig. 2.**
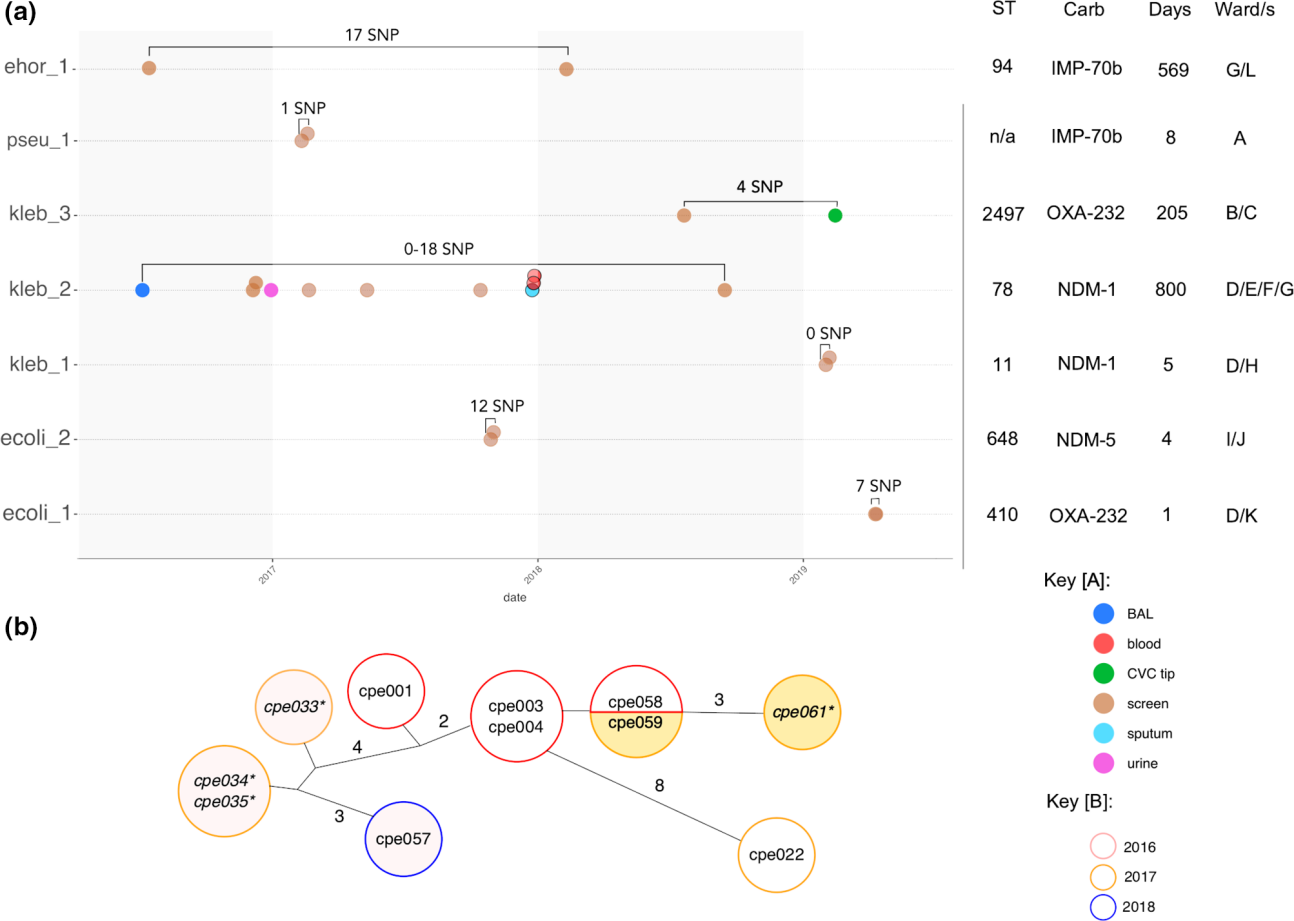
Clonal transmissions identified by WGS: (a) transmission cluster timeline. Species and cluster number provided on the *y*-axis (ehor= *Enterobacter hormaechei *, pseu= *Pseudomonas putida *, kleb= *Klebsiella pneumoniae *, ecoli= *Escherichia coli *). Each row is a different outbreak group, with minimum and maximum core distances between all isolates noted above (as SNPs). The *x*-axis indicates year and approximate date of isolation. Isolates (circles) are coloured by sample site. Isolates outlined in black are duplicate isolates from patients with an existing earlier isolate. BAL=bronchoalveolar lavage, CVC=central venous catheter. The table on the right indicates sequence type (ST), carbapenemase gene, number of days the outbreak persisted (based on available isolates) and the number of affected wards (deidentified). (b) Minimal spanning tree of ST78 *K. pneumoniae* outbreak isolates. Isolates in the same circle were identical at core genome level. Lines represent core SNP distance (1 SNP unless otherwise noted). Circle outlines denote year of isolation. Circle fill colour identifies isolates from the same patient. Isolate names in italics with an asterisk (*) are duplicates isolated from patients already with this *K. pneumoniae* strain.

Enhanced resolution of the carbapenemase genes was also possible from the sequencing data, allowing better discrimination of the *bla*_OXA_ and *bla*_NDM_ alleles (Fig. 2). *bla*_NDM-1_ remained the most prevalent carbapenemase (*n*=19), but this was due to the *bla*_NDM-1_ *K. pneumoniae* outbreak during the study (*n*=11) (detailed below). *bla*_NDM-5_ (*n*=9) was the next most prevalent New-Delhi-metallo-beta-lactamase (NDM) carbapenemase. The *bla*_OXA-48_ group was differentiated into three main types, with *bla*_OXA-181_ (*n*=11) the most prevalent, followed by *bla*_OXA-48_ (*n*=8) and *bla*_OXA-232_ (*n*=8). All other carbapenemase types were found less frequently, with 13/20 (65 %) found in two or fewer isolates. Four isolates that were originally positive for *bla*_NDM_ (cpe078 and cpe020) and *bla*_OXA-48_ (cpe100 and cpe088) based on PCR had no detectable *bla*_NDM_ or *bla*_OXA-48_ after sequencing. This may have been due to loss during subculturing or an error occurring during sample retrieval. These four isolates were not analysed further.

### Variety of carbapenemase alleles and genetic background

**Fig. 3.**
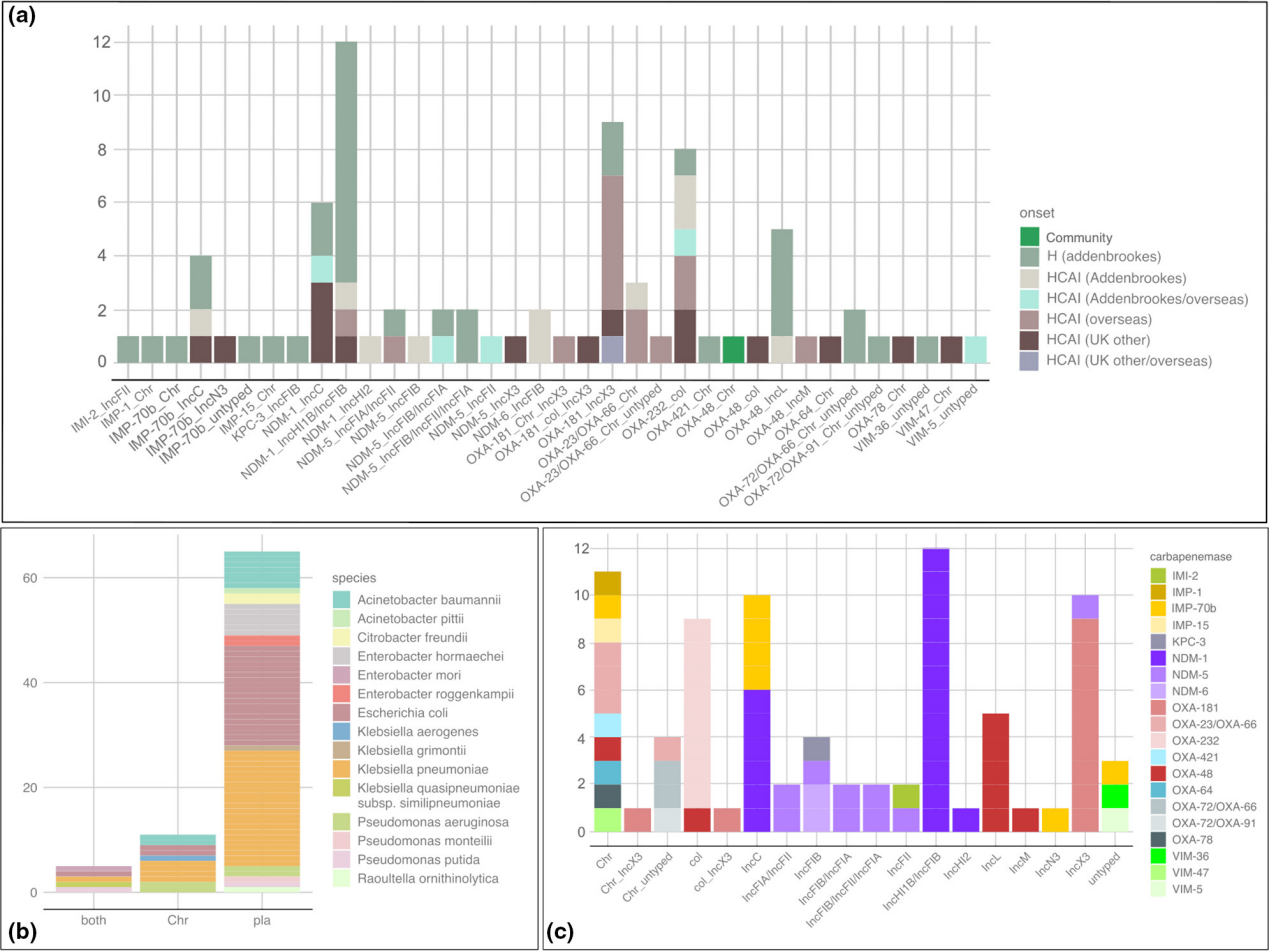
Stacked bar plots of carbapenemase alleles by (a) onset, (b) genomic location and (c) plasmid type. (a) The *x*-axis displays the carbapenemase allele and plasmid replicon type, coloured by onset status (H=hospital onset; HCAI=healthcare-associated) and location of the healthcare association (Addenbrookes=CUH, UK other=UK but non-CUH); (b) the *x*-axis describes whether the carbapenemase gene was found on the chromosome (Chr), a plasmid (pla) or both (coloured by species); (c) the *x*-axis describes location of carbapenemase genes (Chr=chromosome, otherwise specified by plasmid type). Instances of both chromosome and plasmid type indicate where a carbapenemase gene was found on both.

Assessing carbapenemase alleles with their genetic background revealed a wide variety of combinations, which were in many cases unique to a single isolate (Fig. 3a). Further comparison to patient onset information revealed some combinations that appeared more commonly in isolates from patients who had travelled overseas versus those from the UK. These included the IncX3 *bla*_OXA-181_ plasmids, as well as some *bla*_OXA-23_/*bla*_OXA-66_ isolates from *Acinetobacter baumannii* and roughly half of the col *bla*_OXA-232_ isolates. The remainder were all hospital-onset or healthcare-associated in either CUH or other UK hospitals, with the large notable addition of the IncFIB/IncHI1B *bla*_NDM-1_ plasmid that was part of a known outbreak during the study (detailed below). Only one community-associated isolate was sequenced (a chromosomally encoded *bla*_OXA-48_ from an *Escherichia oli*).

### Most carbapenemase genes were located on plasmids

Of 81 isolates, 65 harboured their carbapenemase gene on a plasmid (Fig. 3b). The remaining 16 had their carbapenemase gene on the chromosome (*n*=11) or on both the chromosome and a plasmid (*n*=5). All carbapenemase genes had 100 % nucleotide identity to their assigned allele, with the exception of seven isolates carrying a *bla*_IMP_ gene differing by seven SNPs from *bla*_IMP-1_ and one SNP from *bla*_IMP-70_ (labelled as *bla*_IMP-70b_ in Fig. 3). The single non-synonymous SNP difference from *bla*_IMP-70_ corresponded to I228L.

There was a clear association of certain incompatibility types to carbapenemase genes (Fig. 3c). These include *bla*_OXA-48_ on IncL and IncM, *bla*_OXA-232_ on col, *bla*_NDM-1_ on IncC and IncFIB/IncHI1B, and *bla*_OXA-181_ on IncX3. Several isolates were also identified as harbouring multiple copies of the same carbapenemase gene (details in File S1: Table S1).

### Few clonal transmissions were detected during the study period

Between 2016 and 2020, only seven clonal transmission clusters between different patients were detected from the WGS data (Fig. 2a). The majority of these involved only two patients (*n*=6) and include *P. putida *, *E. coli* (ST410, ST648), *E. hormaechei* (ST94) and *K. pneumoniae* (ST11, ST2497). Most of these outbreaks spanned less than 10 days, with the exception of two ST2497 *K. pneumoniae* that were isolated 205 days apart, and two ST94 *E. hormaechei* isolated 569 days apart. Apart from the *P. putida* isolates, all others were sampled from different wards. Of these events, only the large ST78 *K. pneumoniae* cluster and the *P. putida* cluster were detected by the hospital infection control team at the time. The remainder had no obvious epidemiological connections.

### Outbreak of *bla*_NDM-1_-positive *K. pneumoniae* during the study period

In 2016, a large outbreak of multidrug-resistant *K. pneumoniae* was initially detected during a prospective surveillance study conducted in the adult ICU at CUH. Infection control investigations identified spread to several other wards. WGS identified the outbreak strain as an ST78 *K. pneumoniae *, carrying *bla*_NDM-1_ on a large ~300 kb IncFIB/IncHI1B plasmid. Phenotypic testing found that these isolates (*n*=11) were resistant to a number of antibiotics, including amoxicillin, amoxicillin-clavulanic acid, trimethoprim, ciprofloxacin, cefotaxime, nitrofurantoin, piperacillin-tazobactam, ertapenem, meropenem, fosfomycin, gentamicin, amikacin and aztreonam. They had intermediate resistance to tigecycline and were susceptible to colistin. They carried a number of antibiotic resistance genes, including resistance to aminoglycosides [*AAC(3)-Iid, AAC(6’)-Ib-cr, armA*]*,* cephalosporins (*bla*_CTX-M-15_), quinolones (*qnrB17),* trimethoprims *(dfrA12*) macrolides (*mphE, msrE*), sulphonamides (s*ul1*), and tetracycline [*tet(D)*]. All isolates carried an OmpK36GD porin mutation associated with resistance to carbapenems [49]. Seven isolates had evidence of truncation of *mgrB* (cpe022, cpe003, cpe004, cpe033, cpe034, cpe035) or *pmrB* (cpe001) associated with resistance to colistin (despite phenotypic susceptibility). Four of these also carried aerobactin *iuc1* (cpe004, cpe022) or *iuc5* (cpe001, cpe003), but no other genetic markers for hypervirulence were observed [50].

Based on the WGS data obtained here, this outbreak involved at least seven patients from July 2016, with one patient testing positive again with the same organism in 2018 despite being sent home (Fig. 2b). As not all isolates from the study period were sequenced, it is also possible that other non-sequenced isolates from our dataset were also part of the outbreak (File S1: Fig. S8).

To provide further context to the outbreak, we interrogated a large public repository of 661 405 bacterial genomes collated up to 2018, which identified 352 broadly related isolates. Only 28 of these were identified as ST78, while the vast majority were found to be ST14, a single-locus variant of ST78 (File S1: Table S2).

**Fig. 4.**
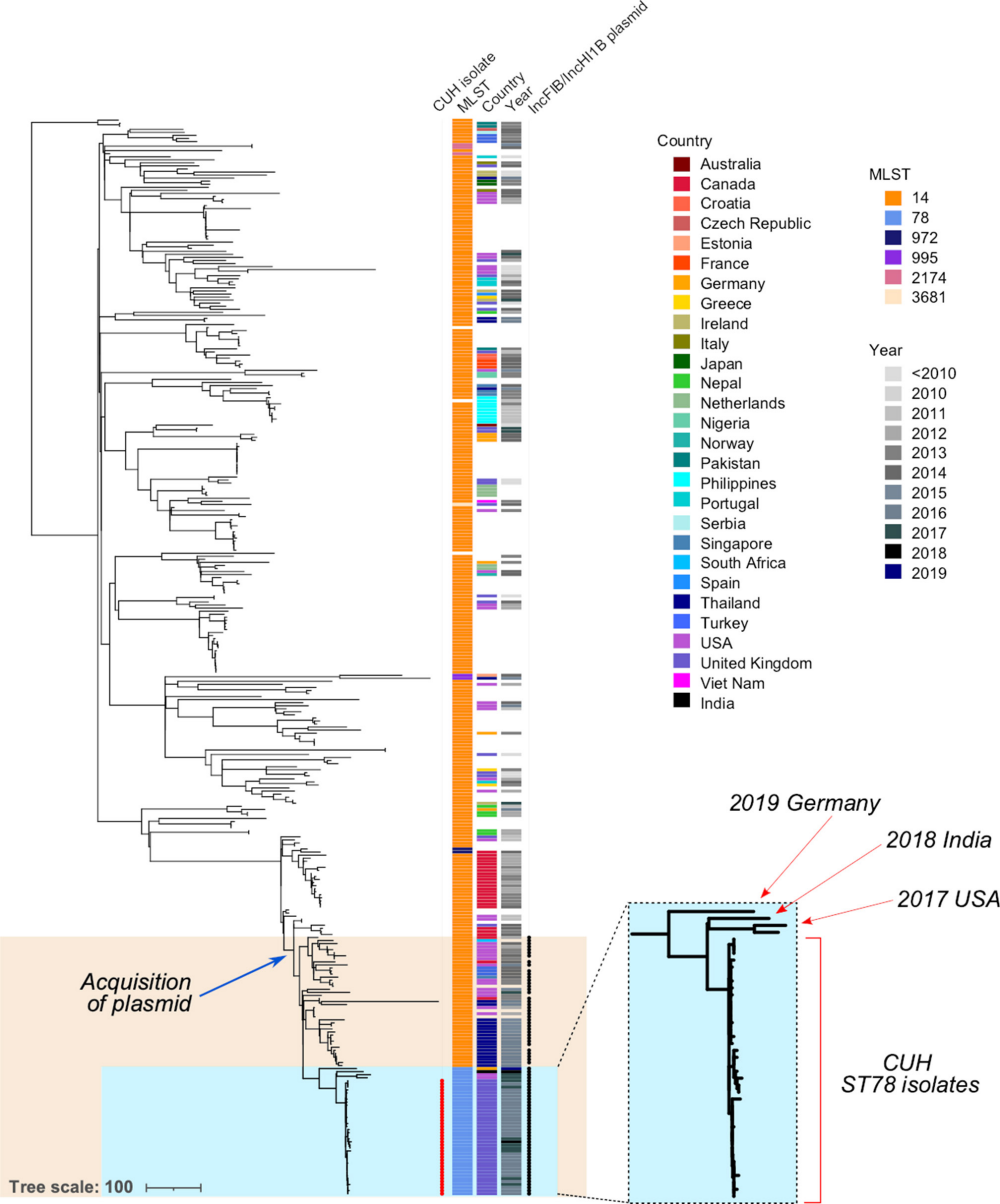
Phylogeny of ST78 outbreak strains and closest relatives from a repository of ~661 000 bacteria. Blue box: all available ST78 isolates, mainly isolated from CUH with four external ST78 isolates. Orange box: all isolates from this collection that harbour the IncFIB/IncHI1B plasmid carrying *bla*_NDM-1_, acquired in an ST14 ancestor to the ST78 clade. Bar, number of substitutions.

Surprisingly, all of the available ST78 strains (*n*=28) from the public repository were also isolated at CUH and obtained in 2016 or 2017 as part of further intensive screening of the outbreak (Fig. 4, blue box). Only one isolate appeared to be a duplicate that was sequenced in both this study and a previous study (cpe001; ER2672819). As we did not expect to find only CUH isolates, we also downloaded all available *K. pneumoniae* assemblies from NCBI (11 811 sequences) and performed *in silico* MLST. None of these additional assemblies were found to be ST78. We then performed a literature search, which uncovered a single reported ST78 *K. pneumoniae* isolate from India in 2018 [51] which we then added to our analysis. We also inspected Pathogenwatch and found three additional isolates (two from the USA and one from Germany). All four additional isolates were positioned outside of the main ST78 outbreak clade in the phylogenetic tree, suggesting that they are indirectly related through a distant relative. Phylogenetic comparison to other contextual strains revealed that the closest relatives were of ST14, mainly from Thailand, the USA and Canada (Fig. 4). The index patient identified in CUH had no history of travel or hospitalization abroad in the 3 months prior to admission.

We were also able to search the same public repository for isolates with a high likelihood of carrying the *bla*_NDM-1_ IncFIB/IncHI1B plasmid based on >80 % matching kmer identities. We found 173 isolates all belonging to *K. pneumoniae* from a variety of STs, the majority being ST15, ST14 and ST78 (File S1: Fig. S9). The isolates with the highest identity to our plasmid were all from ST14 and ST78. We checked for the presence of *bla*_NDM-1_ in these isolates and found that 141 (82 %) carried the gene, which was found 100 % of the time in isolates with a kmer identity ≥95 %.

We then compared our plasmid results to our contextual isolates (Fig. 4) to identify those that carried the IncFIB/IncHI1B plasmid, with or without *bla*_NDM-1_. We found that only those strains closely related to the ST78 outbreak carried this plasmid, including the four non-CUH ST78 isolates (Fig. 4 **,** orange box;File S1: Fig. S10). Of these 65, only five did not carry a *bla*_NDM_ gene. All other carried *bla*_NDM-1_. As such, acquisition of this plasmid probably occurred once in an ancestral ST14 isolate and has been stably maintained with infrequent loss (Fig. 4).

### Combination of clonal and horizontal plasmid transmission, mixed with high background variability

In total, 70 isolates (86%) were found to carry a carbapenemase gene on a plasmid. By comparing plasmid sequences from these isolates and using a mash distance threshold of 0.001, we identified 44 isolates (from 31 patients) that had carbapenemase plasmids that clustered closely. These 44 isolates spanned 10 plasmid clusters (Table 2). Of the remaining 26 isolates, five formed clusters when applying a less strict threshold (0.005). The other 21 remained unique, resulting in a total of 31 plasmid backgrounds over the study period.

**Table 2.** Carbapenemase plasmid clusters

Cluster	No. of isolates	Species	Patients	Carbapenemase gene	Plasmid type
**Intra-patient transmission**					
Clust_217	4	*Klebsiella grimontii, Enterobacter roggenkampii, Raoultella ornithinolytica*	1	OXA-181	IncX3
Clust_33	2	*Escherichia coli, Klebsiella pneumoniae*	1	NDM-6	IncFIB
Clust_148*	4	*Escherichia coli, Klebsiella pneumoniae*	2	NDM-1	IncC
**Clonal transmission**					
Clust_4	9	* Klebsiella pneumoniae *	6	NDM-1	IncHI1B/IncFIB
**Horizontal transmission**					
Clust_133	2	* Acinetobacter baumannii *	2	OXA-72	Untyped
Clust_17		*Klebsiella aerogenes, Escherichia coli, Klebsiella pneumoniae*	3	OXA-48	IncL
Clust_54	2	* Escherichia coli *	2	NDM-5	IncFIB/IncFIA
Clust_72*	8	*Klebsiella pneumoniae, Escherichia coli*	8	OXA-232	Col
Clust_9*	3	* Enterobacter hormaechei *	3	IMP-70b	IncC
Clust_98	6	*Escherichia coli, Enterobacter hormaechei, Klebsiella pneumoniae*	6	OXA-181	IncX3

*Contains some clonal transmission of bacterial strains in addition to horizontal transfer.

Broadly, there were three mechanisms identified in this dataset that explained why closely related plasmids were identified across multiple isolates: (i) *clonal transmission*, i.e. a bacterial strain infects or colonizes multiple hosts carrying the same plasmid; (ii) *intra-patient transfer*, where a plasmid has moved from one bacterial host to another within a single patient; and (iii) *horizontal transfer* between different bacterial species/strains detected outside of a single patient (Table 2). Two clusters (clust_217 and clust_33) appeared to be the result of intra-patient plasmid transfer between different colonizing species, as they were exclusive to a single patient. Two patients in cluster 148 (clust_148) appear to share the same ST11 *K. pneumoniae* strain but different *Escherichia coli (*ST10 and ST58), suggesting the *K. pneumoniae* transmitted between the patients first, after which the plasmid was transferred into different *Escherichia coli* strains in either patient. Cluster 4 (clust_4) was entirely the result of clonal transmission of the ST78 outbreak *K. pneumoniae *, rather than independent plasmid transfer. The largest cluster (clust_72) contained four clonally related isolates (two *K. pneumoniae* and two *Escherichia coli *) alongside another four unrelated strains that all harboured a *bla*_OXA-232_-col plasmid. Based on this analysis, out of 70 isolates, 22 (31 %) carried plasmids with carbapenemase genes shared across different patients and as a result of historical or recent horizontal transmission (and not intra-patient transfer of the plasmid between hosts or clonal dissemination).

Looking across all plasmids in the dataset and clustering at the same mash threshold (0.001), we found a total of 51 clusters ranging from 2 to 11 isolates in size (median=2). The largest clusters were other plasmids associated with the ST78 *K. pneumoniae* outbreak strain (i.e. the result of clonal transmission). These were followed by the *bla*_OXA-181_ and *bla*_OXA-232_ plasmid clusters, as described above. There were no other non-carbapenemase-carrying plasmids that appeared highly prevalent across isolates.

There was limited evidence of smaller mobile genetic elements (MGEs) involved in horizontal transmission when examining the context of the carbapenemase genes in this dataset (data not shown). As such, we found that plasmids were the main vehicle of carbapenemase transmission in this study, both vertically (clonally) and horizontally.

## Discussion

This study conducted a detailed analysis of carbapenemase-producing Gram-negative bacteria in a single UK hospital over 6 years. Overall, we found low rates of carbapenemase-producing organisms, and this did not appear to increase substantially during the study period. This is consistent with a previous point prevalence study, conducted in the same hospital, which found no CPE amongst 540 screening samples collected over a 3 day period in 2017 [52].

Additionally, we found very little evidence for the circulation of dominant lineages, and instead identified a variety of species carrying a range of carbapenemase alleles in different genetic backgrounds. Previous healthcare exposure (mainly at CUH) was a common feature among patients in this study and could be concluded as the main risk for infection with carbapenemase-producing bacteria. Indeed, a previous study looking at carbapenem-resistant *K. pneumoniae* across Europe found high levels of within-hospital spread [40]. However, if these were hospital-associated lineages, we might expect to see more frequent or consistent transmission over the 6 year timeframe, but instead we found limited transmission beyond a single patient.

Another possibility is independent introduction of these strains via patient carriage as a result of prior acquisition in other healthcare or community settings, which would explain the limited clonal transmission and high diversity we have observed. Carriage of carbapenemase genes is not uncommon, and can persist for prolonged periods of time, particularly following antibiotic exposure in hospitals [53, 54]. To verify this, we would need substantially more contextual data from the community and other healthcare settings. These areas represent gaps in our understanding of carbapenemase-producers nationally.

The few clusters that were detected during this study rarely exceeded two patients and most had no epidemiological support other than close temporal links within the same institution. This could indicate that transmission occurred in the hospital via an intermediate, such as contaminated surfaces or hospital staff [55]. Despite these seemingly benign transmissions, these cryptic events do reveal how easily bacteria can begin to spread undetected and in the absence of other epidemiological links. Early detection of spread is crucial to guide infection control interventions, and it can also have an enormous impact on healthcare costs [56]. Routine use of WGS provides a means to detect transmission early, but the costs and logistics have hampered routine adoption in healthcare settings [57]. Even in this study, we were unable to sequence all carbapenemase-producers, limiting our ability to investigate the relatedness of unsequenced isolates.

An easily searchable index of public data is crucial for outbreak analysis and public health interests. Blackwell *et al.* [44] provide a searchable index of all bacterial isolates from the ENA up to 2018, which enabled us to rapidly contextualize our ST78 *K. pneumoniae* outbreak strain. Coupled with all *K. pneumoniae* assemblies submitted to NCBI, we observed almost no publicly available ST78 isolates outside of those obtained at CUH, and only a few distant relatives [51]. While we do not have ENA data submitted after 2018, we would expect public data for ST78 to have existed prior to this if it had been reported elsewhere since the ST78 outbreak strain first appeared in CUH in 2016. Here we determined that this strain is extensively resistant to antibiotics but mostly lacking biomarkers for hypervirulence. Vigilance and further surveillance is required to monitor this ST and its possible expansion to other parts of the UK.

Finally, we used long-read sequencing to analyse plasmids in this study. Short-read sequencing is usually insufficient to confidently identify and compare plasmid sequences from whole genomes. Here, we were able to determine the number of shared and unique carbapenemase-carrying backgrounds between isolates, the majority of which were on plasmids. We found several plasmids of importance that have been identified elsewhere, including pOXA-48 [58], col_OXA-232 [59] and IncX3_OXA-181 [60]. By determining which isolates were related clonally, we could infer which plasmids were more likely to have been transmitted horizontally. However, we were unable to confidently predict whether horizontal plasmid transfer had occurred during or prior to hospitalization. Plasmids do not appear to follow the same mutation rate expected from clonally spreading strains, as evidenced by comparisons of pOXA-48 [61]. As such, distantly transferred plasmids can still have very few SNP differences. We also do not have access to susceptible strain information that could be used to identify the patient’s isolate prior to plasmid acquisition.

We acknowledge several limitations to our study. First, the hospital screening policy and laboratory diagnostic methods for detection of carbapenemase-producing organisms changed over the course of the study period. This may have resulted in an underestimate of the true prevalence of these organisms, and/or an overestimation of hospital onset, since not all patients were screened on admission. Second, not all samples were stored in the diagnostic laboratory and/or available for sequencing. Third, we did not sample healthcare workers and did not routinely sample the environment as part of this study. Fourth, there was limited sampling (and associated metadata) outside the hospital setting. All of these factors may have affected our ability to detect and analyse all potential transmission events.

Nevertheless, we found considerable diversity of species in our dataset with minimal transmission and largely from carriage isolates. Our results suggest that these isolates may represent independent introductions into CUH. Detailed resolution using WGS and contextualization with public data allowed us to better construct clonal and plasmid relationships, particularly for an emerging extensively resistant ST78 *K. pneumoniae* lineage. However, a national framework to collate contextual data, particularly for plasmids and resistant bacteria broadly in the community, is needed to better understand how carbapenemase genes are transmitted in the UK.

## Supplementary Data

Supplementary material 1
