# Supplementary material for: Long-read sequencing reveals genomic diversity and associated plasmid movement of carbapenemase-producing bacteria in a UK hospital over 6 years
Source: Microb Genom. 2023 Jul 5;9(7):mgen001048. doi: 10.1099/mgen.0.001048 (PMC10438816; doi:10.1099/mgen.0.001048)
Supplement: Supplementary material 1 [file mgen-9-1048-s001.pdf]

SUPPLEMENTARY MATERIALS

Additional File 1: Supplementary Methods and Results

1. **Supplementary Methods**
2. **Supplementary Results**
3. **Supplementary Tables**
    1. Additional information about study isolates harbouring duplicate carbapenemase genes.
    2. Additional information about sequence types of study isolates.
4. **Supplementary Figures**
    1. counts of pairwise SNP distances between putative transmission pairs (calculated using Snippy)
    2. Bacterial species and carbapenemase genes stratified by isolate source.
    3. Species and carbapenemase genes stratified by infection type.
    4. Meropenem resistance vs. species and carbapenemase genes
    5. Timeline of study samples showing bacterial genus (panel A), carbapenemase genes (panel B), Sequenced samples (blue) and evidence of transmission (black box) (panel C), and genomic clusters and species (panel D).
    6. CUH bed occupancy compared to admitted patients with CPE
    7. Bacterial species and carbapenemase genes classified by travel history.
    8. Study timeline showing sequenced NDM-positive isolates and non-sequenced isolates. Different species indicated by different colours
    9. Isolates found to have >0.8 (80%) kmer identity to the outbreak IncFIB/IncHI1B plasmid
    10. BRIG image of Indian ST78 isolate compared to IncFIB/IncHIB NDM-1+ plasmid
5. **References**

Additional File 2: Sample Metadata and Accession numbers

1. **Table 1: Sample metadata**
2. **Table 2: Sample accession numbers**

Supplementary Methods

CPE Screening policies:

CPE screening polices within CUH changed during the course of the study. In 2014 CPE screening was based on risk factors as defined in the Public Health England guidance (1). In summary, patients who had been admitted to a hospital overseas or London / northwest England in the preceding year were screened on admission (three times, 48 hours apart). Screening was increased in 2016 following an outbreak of CPE to include all admissions to adult intensive care units (ICUs), with weekly screens thereafter (see below). Certain high risk patient populations were also screened on admission or prior to admission (e.g. multivisceral transplant patients and patients referred for surgery due to encapsulating peritoneal sclerosis). Screening was also instigated at the discretion of the infection prevention & control team (IPCT) when a transmission event / outbreak was suspected. Screening criteria for multi-resistant *Acinetobacter* spp. did not change over the study period; it included patients admitted to hospital abroad in the preceding year, or at the discretion of the IPCT. Screening for carbapenem-resistant *Pseudomonas* spp. did not occur.

In 2014, screening samples to detect CPE were plated onto Brilliance ESBL chromogenic agar (Oxoid Ltd, Basingstoke UK). Screening samples to detect multi-resistant *Acinetobacter* spp. were inoculated onto Brilliance UTI Chromogenic agar and CLED agar (Oxoid Ltd, Basingstoke UK). Bacterial species were identified using MALDI-TOF MS (Bruker Diagnostics, Bremen, Germany). Susceptibility testing was performed using British Society for Antimicrobial Chemotherapy (BSAC) guidelines until April 2018, and European Committee on Antimicrobial Susceptibility Testing (EUCAST) thereafter. Isolates with suggestive phenotypes were sent to Antimicrobial Resistance and Healthcare Associated Infections (AMRHAI) national reference laboratory for confirmation and formal typing. In January 2017, COLOREX mSuperCARBA selective chromogenic agar (E&O Laboratories, Bonnybridge, UK) was introduced to detect CPE. Cepheid Xpert® Carba-R (Cepheid, Sunnyvale, USA) was introduced in-house to confirm the presence of the 5 commonest carbapenemases in the UK (bla_{KPC}, bla_{NDM}, bla_{OXA-48}, bla_{IMP}, bla_{VIM}). Isolates with high suspicion for carbapenemase production based on EUCAST screening guidance (2) and *Acinetobacter* spp. were still referred to AMRHAI for confirmation of carbapenemases not included in the Cepheid Xpert® Carba-R panel.

Onset definitions:

Isolates were described as hospital-onset (HAI) if detected more than 48 hours after admission of the patient. Healthcare-associated (HCAI) isolates were isolates detected within 48 hours of admission and where the patient had been hospitalised in the preceding 12 months. Community-associated (CAI) isolates were from admitted patients with no hospitalisation in the last 12 months and isolated within 48 hours of admission, or from persons presenting to general practices (GPs) with no hospitalisation in the year prior.

Illumina and ONT sequencing:

For short-read sequencing, 150-base paired libraries were prepared according to standard protocols and sequenced on the Illumina HiSeq platform at the Sanger Institute. Isolates were also long-read sequenced using a MinION device and R9.4.1 flow cells (Oxford Nanopore Technologies (ONT), Oxford, UK). The majority were multiplexed (between 3-12 isolates per flow cell) using the SQK-RBK004 rapid barcoding kit. The exception were three isolates (cpe012, cpe023, cpe026) which were multiplexed using the SQK-LSK108 ligation kit with the ND103 native barcoding kit.

Nanopore basecalling, demultiplexing and filtering:

All fast5 reads were basecalled and demultiplexed with Guppy (v3.6.0), using the "high-accuracy" basecalling model and the relevant barcoding kit for demultiplexing. Basecalled reads were then filtered for quality using Nanofilt (v2.7.1) (3) at a threshold of Q7 and a minimum read length of 1000 base pairs (bp). Isolates with >110x coverage were additionally subsampled down to 100x coverage using Filtlong (v0.2.0) (with --keep_percent 90 and --target_bases *corresponding to 100x coverage for that isolate*) (4).

Nanopore assembly and polishing:

All isolate Nanopore reads (original and subsampled) were *de novo* assembled using Flye (v2.8) (with --plasmids flag) (5), Unicycler (v0.4.8) (hybrid settings) (6) and Canu (v2.1.1) (default) (7). Samples with high coverage (>110x cov) were subsampled down to 100x coverage using Filtlong (0.2.0) to try and improve assembly. Based on "completeness" (measured by an intact chromosome and circularised contigs), the best assembly per isolate was selected, with a preference for Flye > Unicycler > Canu

when the assemblies were comparable. Isolates with no good quality assembly were further processed using Circlator (v1.5.5_py2) (8) with the Canu corrected reads. Final assemblies for each isolate were polished using NextPolish (v1.3.1) (9) and the Illumina trimmed reads. Assembly metrics are provided in **Additional File 2**.

Comparison to public data:

The genome of ST78 *K. pneumoniae* strain cpe058 was sketched using sourmash (v3.5.0) (10) at a Kmer length of 31 bp and an interval of 5000 bp ("-n 5000 -k 31") and was used as a query against the sourmash index of 661,405 bacterial genomes (11). The estimated core distances of the 2,000 sample accessions with the highest similarity to cpe058 was extracted from the pp-sketch index of the 661K using pp-sketchlib (v1.5.1) (12). A neighbour-joining tree of the core distances was produced using RapidNJ (v2.3.2) (13) and the tree and was visualised in R using ggtree (v3.0.1) (14). By layering the similarity of the genomes to that of the cpe058 genome onto the phylogeny, the clade containing the most closely related genomes could be identified. This clade contained 352 genomes and the metadata and additional characterisation data was extracted from figshare (11). 350 isolates with Illumina reads were downloaded from the ENA.

In addition to the 661K database, we also downloaded all *K. pneumoniae* assemblies from NCBI (11,811, accessed 31-August-2021). We then used mlst (v2.19.0) to determine sequence types for all assemblies.

A single isolate obtained in India (NCBI: NZ_JAJBIZ010000100.1) was identified during a literature search for ST78 *K. pneumoniae* (15). As the reads were not provided, we simulated reads using ART_Illumina (version 2.5.8) (16) from the draft assembly available on NCBI (accessed November 29th 2021) with the following flags: "--noALN --seqSys HS25 --len 150 --fcov 50 --mflen 500 --sdev 25".

We also searched for the ~300 kb *bla*$_{NDM-1}$ IncFIB/IncHI1B plasmid in the 661k database. pCPE058 (IncFIB/IncHI1B plasmid with *bla*$_{NDM-1}$) was used to query the COBS index (17) of 661K genomes (11) with a kmer similarity cut-off of 0.8.

Supplementary Results

Duplicate carbapenemase genes identified in isolates:

Three *A. baumannii* isolates (cpe011, cpe044 and cpe045) carried several copies of *bla*$_{OXA-23}$ in separate Tn*2006* elements at different loci. Two *E. coli* were found to carry two *bla*$_{OXA-232}$ genes as a result of a col plasmid dimer (cpe086, cpe092). *bla*$_{OXA-181}$ was found twice in cpe106 (*E. roggenkampii*) as a result of the same IS*26* composite transposon found on both the chromosome and the plasmid. Cpe005 (*P. monteilii*) carried tandemly duplicated *bla*$_{IMP-15}$ in an ICE element closely related to ICEPaeCB5. Similarly, cpe019 (*P. aeruginosa*) carried two *bla*$_{VIM-47}$ cassettes in a Tn*402*-like integron similar to Tn*6352*. Finally, a *K. pneumoniae* isolate (cpe001) appeared to carry between 2 to 6 copies of *bla*$_{NDM-1}$ tandemly duplicated within an ~11 kb region on a large (~360 kb) IncFIB/IncHI1B plasmid. This region appeared to be flanked by IS*Aba125*, appearing in a single copy in other related IncFIB/IncHI1B plasmids from this dataset and in a related reference plasmid KpST14-NDM-1 (GenBank: CP071280.1). The exact number of copies could not be determined as there were no reads that traversed the entire length of the duplicated region.

Supplementary Tables

Supplementary Table 1. Additional information about study isolates harbouring duplicate carbapenemase genes.

Isolate	Species	carbapenemase	Description
Cpe005	*Pseudomonas monteilii*	IMP-15	Inside ICE element, closely related to ICEPaeCB5 (Tn6391) with additional IMP-15 tandem duplication, aacA4, aadB, and ant1 genes
Cpe019	*Pseudomonas aeruginosa*	VIM-47	A Tn402-like integron, similar to Tn6352, with a ISPa7 replacing the ISPa17, and tandem duplication of VIM-47 and aacA4 cassettes replacing AAC(6')-II and AAC(3)-I cassettes.
Cpe086	*Escherichia coli*	OXA-232	Col plasmid dimer resulting in complete duplication of the plasmid
Cpe092	*Escherichia coli*	OXA-232	
Cpe106	*Enterobacter roggenkampii*	OXA-181	1 on chromosome and 1 on plasmid: ~10 kb composite transposon: Tn3 family IS3000 upstream, flanked by IS26
Cpe045	*Acinetobacter baumannii*	OXA-23/OXA-66	Two copies of Tn2006 at different loci
cpe011	*Acinetobacter baumannii*	OXA-23/OXA-66	1 copy of Tn2006, 2 copies of Tn2006-like element, that both have inverted duplicate copies of OXA-23 flanked again by IS26 elements (5 copies total)
Cpe044	*Acinetobacter baumannii*	OXA-23/OXA-66	4 copies of Tn2006 carrying OXA-23 at different loci
Cpe001	*Klebsiella pneumoniae*	NDM-1	Tandem duplication of NDM-1 at least twice; Nanopore read length insufficient to determine true number of copies. Illumina read coverage supports up to 6 copies.

Supplementary Table 2. Additional information about sequence types of study isolates.

ST	gapA	infB	mdh	pgi	phoE	rpoB	tonB
14	1	6	1	1	1	1	1
15	1	1	1	1	1	1	1
78	1	6	1	1	1	9	1

Supplementary Figures

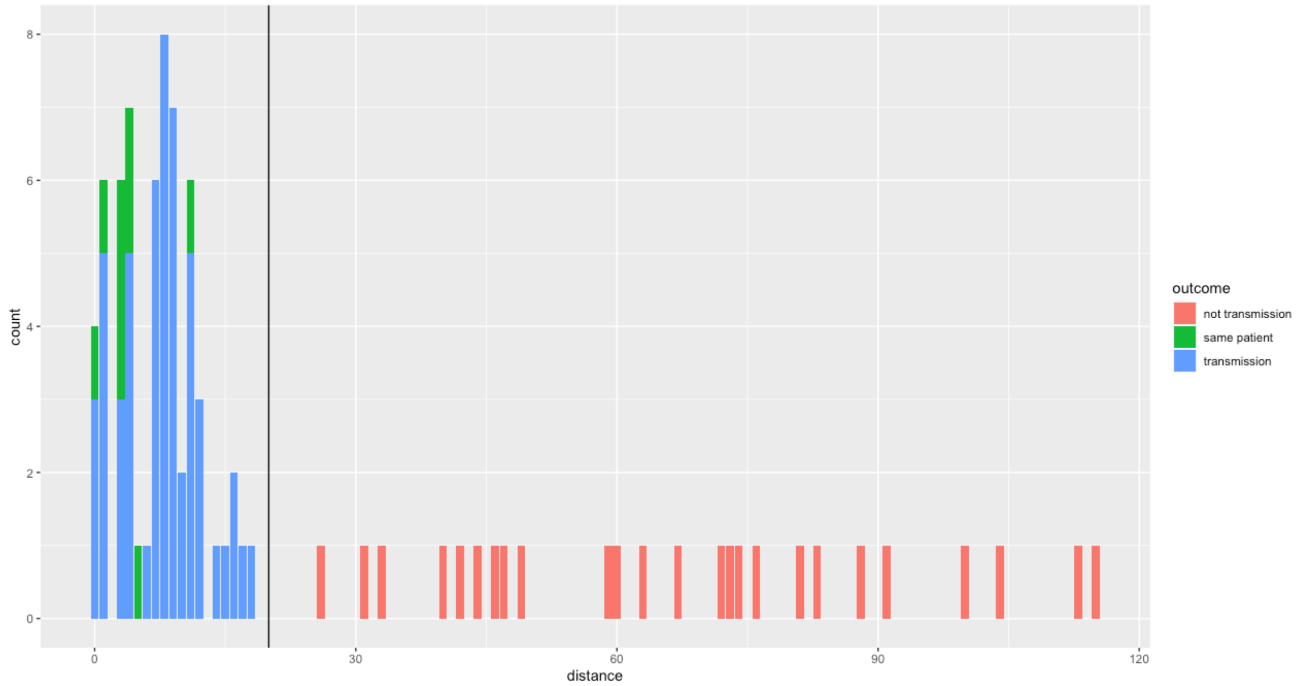

Supplementary Figure 1: counts of pairwise SNP distances between putative transmission pairs (calculated using Snippy): all putative paired isolates were compared using Snippy and an internal reference genome to calculate single nucleotide polymorphism (SNP) distances. Y-axis = counts, x-axis = SNP distance. The vertical black line represents the maximum SNP cutoff for transmission (20 SNPs). Pairwise SNP distances are coloured based on our evaluation (red = not a transmission pair, green = pair from same patient, blue = putative transmission).

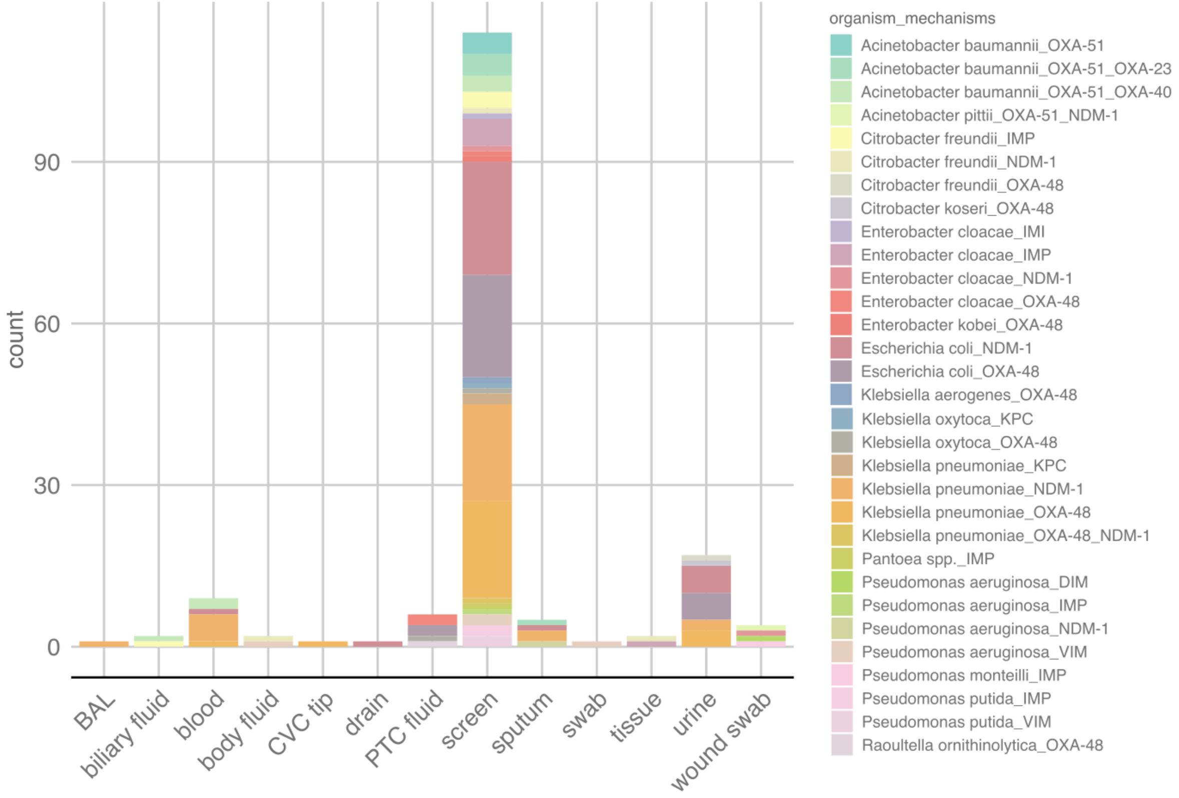

Supplementary Figure 2: Bacterial species and carbapenemase genes stratified by isolate source. BAL = bronchoalveolar lavage, CVC = central venous catheter, PTC = Percutaneous transhepatic cholangiography.

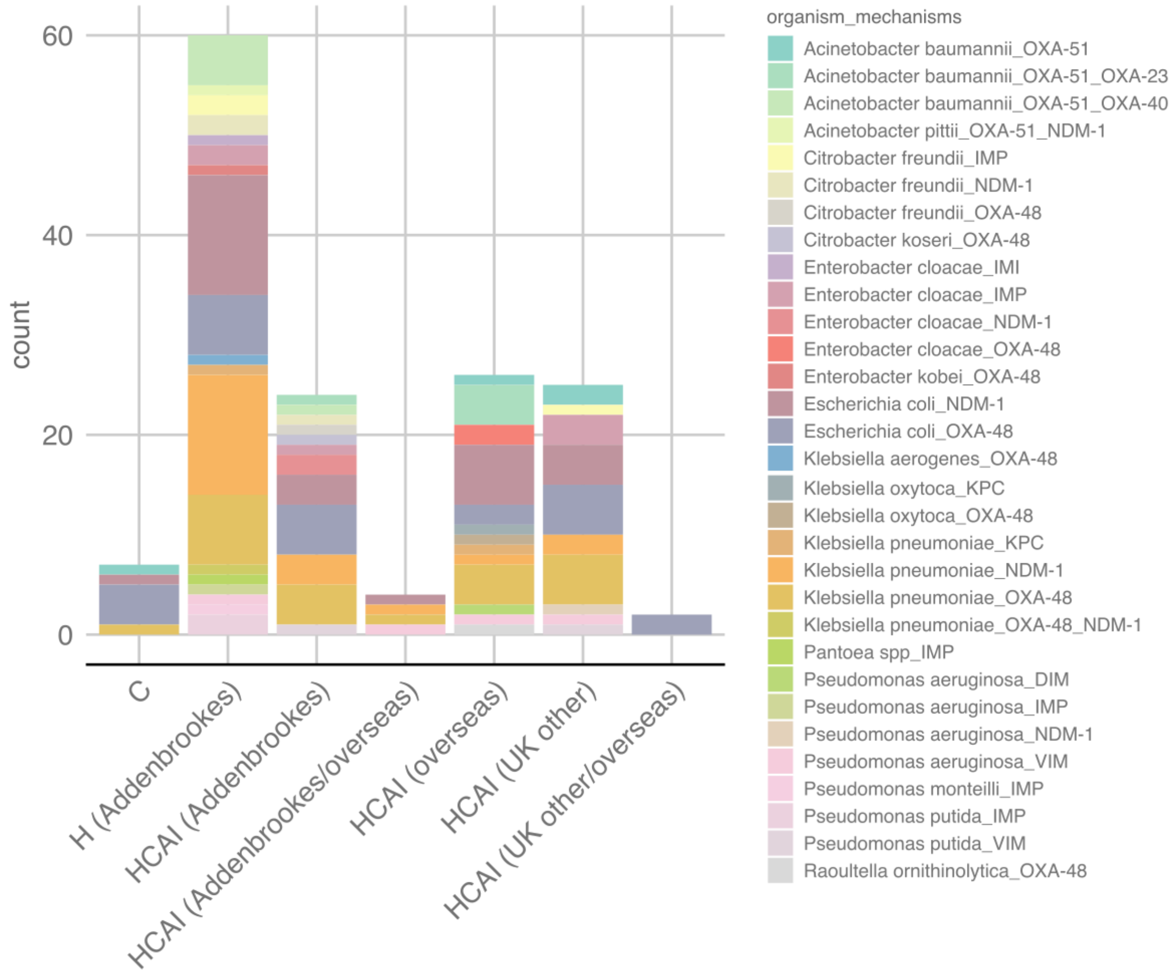

Supplementary Figure 3: Species and carbapenemase genes stratified by infection type. C = community-associated; H = hospital-onset; HCAI = healthcare-associated; UK = United Kingdom.

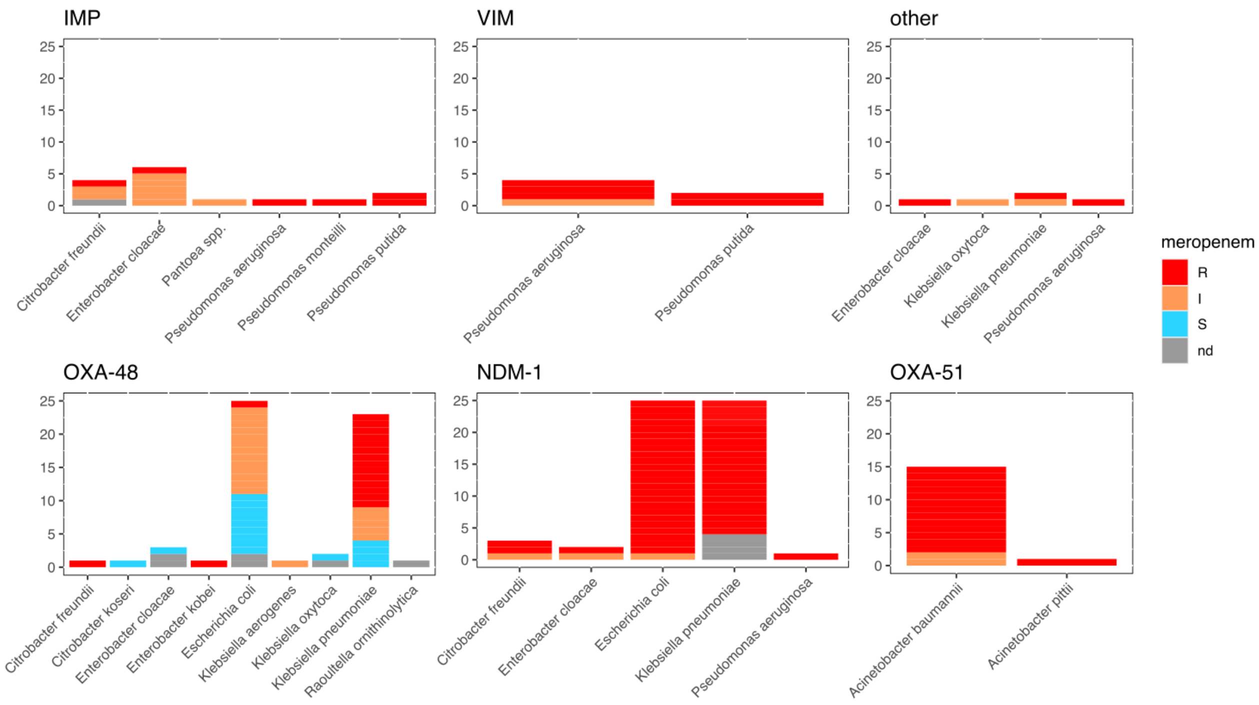

Supplementary Figure 4: Meropenem resistance vs. species and carbapenemase genes. Colours indicate resistance level: blue = S (sensitive), orange = I (intermediate), red = R (resistant). Samples with no data (nd) were coloured grey. Other = DIM+ *Pseudomonas aeruginosa* (n=1), KPC+ *Klebsiella* sp. (n=3), IMI+ *Enterobacter cloacae* (n=1).

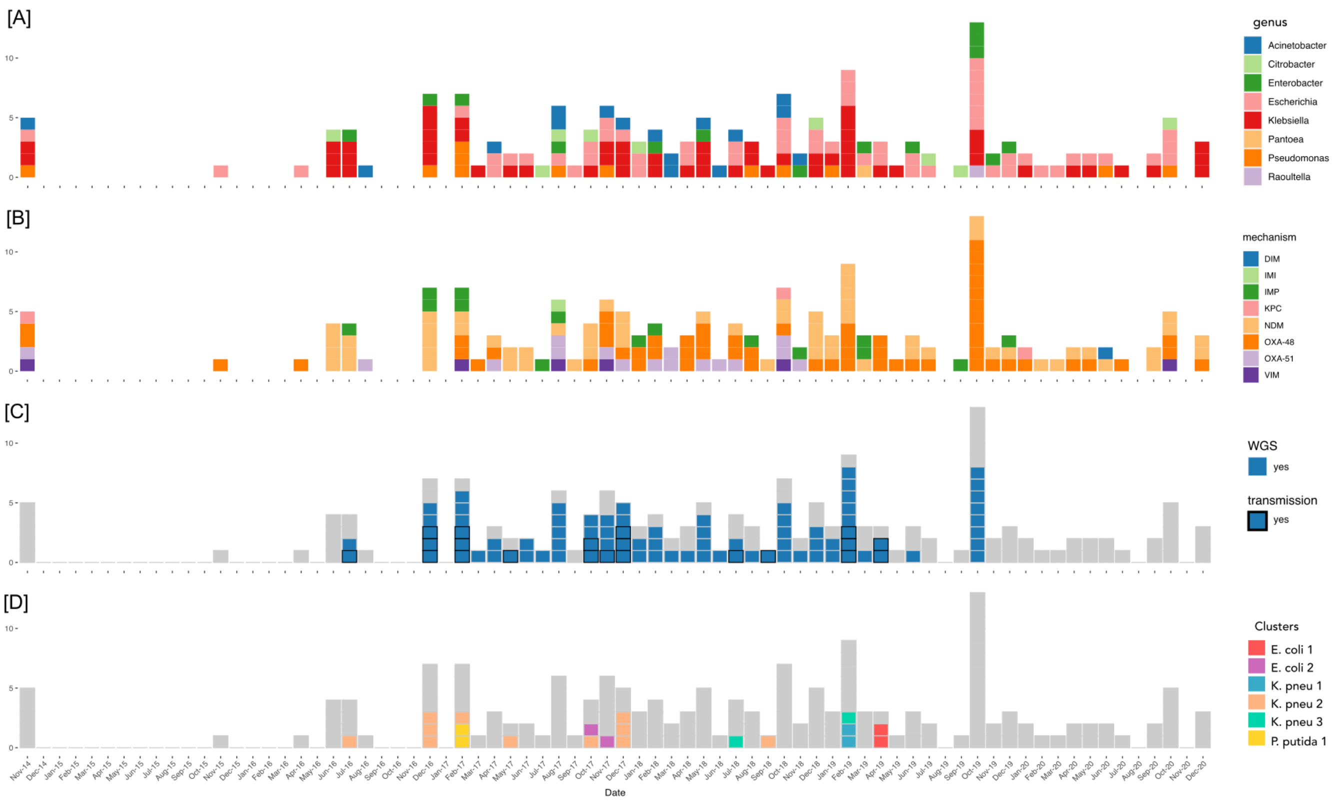

Supplementary Figure 5: Timeline of study samples showing bacterial genus (panel A), carbapenemase genes (panel B), Sequenced samples (blue) and evidence of transmission (black box) (panel C), and genomic clusters and species (panel D).

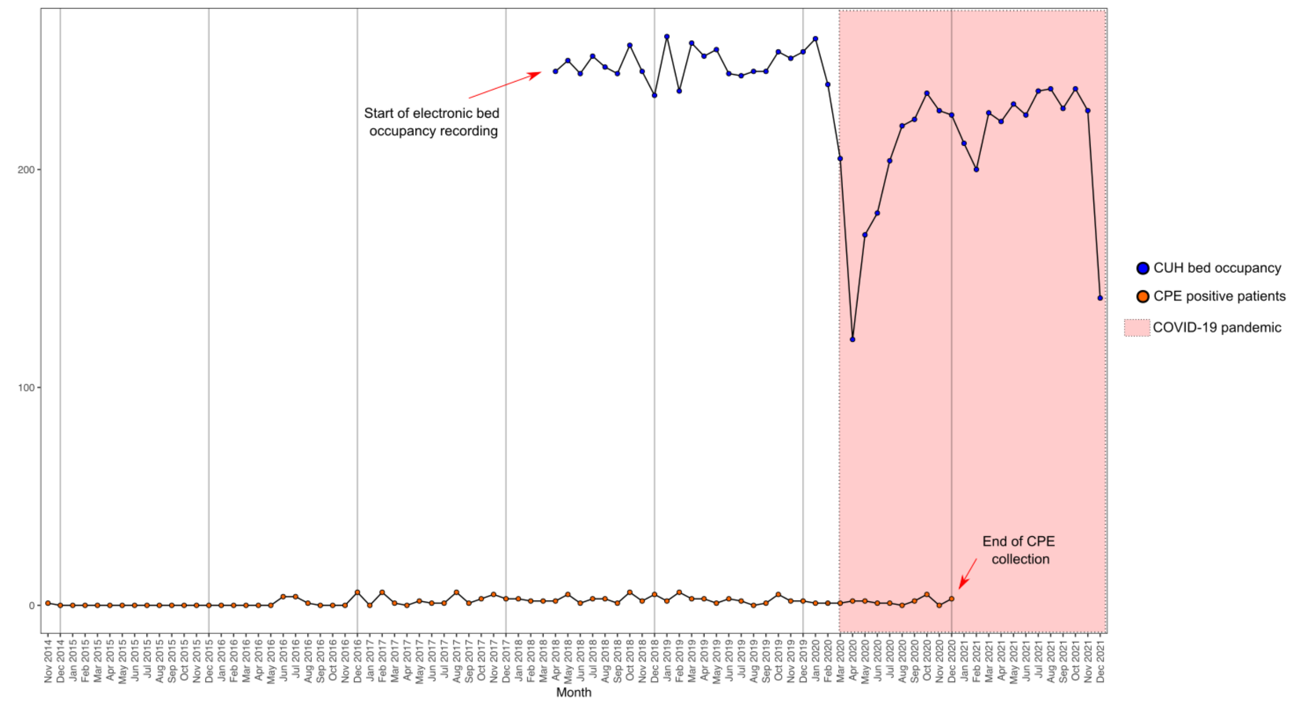

Supplementary Figure 6: CUH bed occupancy compared to admitted patients with CPE: Y-axis = month and year. X-axis = patient counts. Line with blue dots: CUH bed occupancy per month from April 2018 (start of electronic recording). Counts have been scaled down 100x to fit figure. Excludes maternity, mental health and births. Black line with orange dots: number of patients with CPE isolates per month (excludes GP cases from study). Collection ended in December 2020. Red shaded area: COVID-19 pandemic (March 2020 onwards).

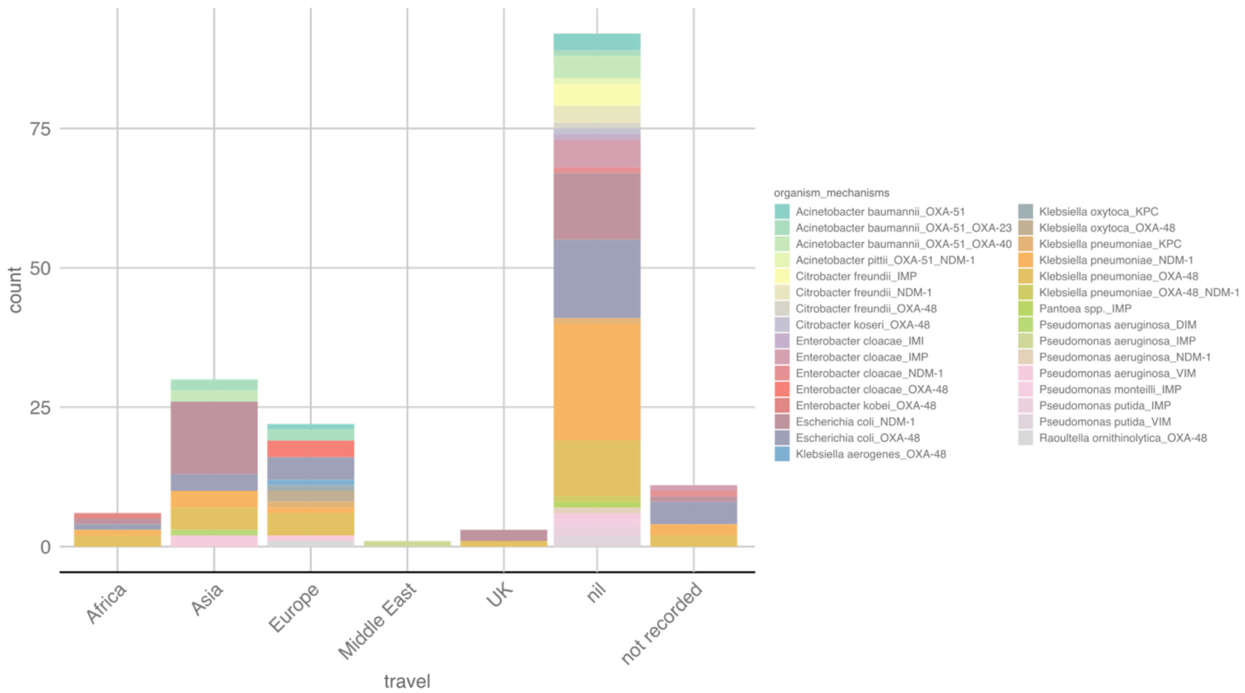

Supplementary Figure 7: Bacterial species and carbapenemase genes classified by travel history.

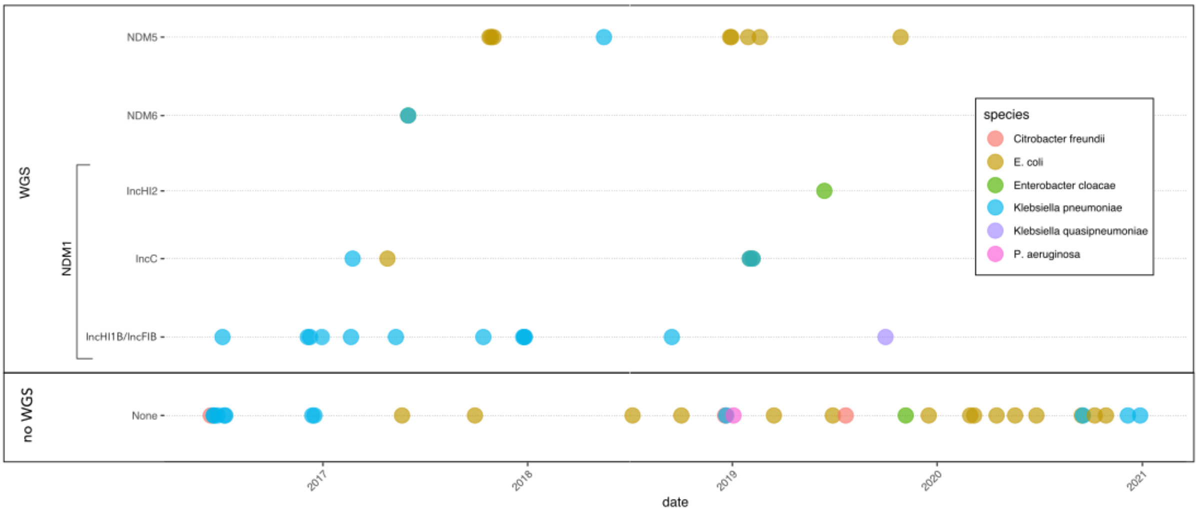

Supplementary Figure 8: Study timeline showing sequenced NDM-positive isolates and non-sequenced isolates. Different species indicated by different colours. Background colour indicates whether WGS data was available for the isolate or not.

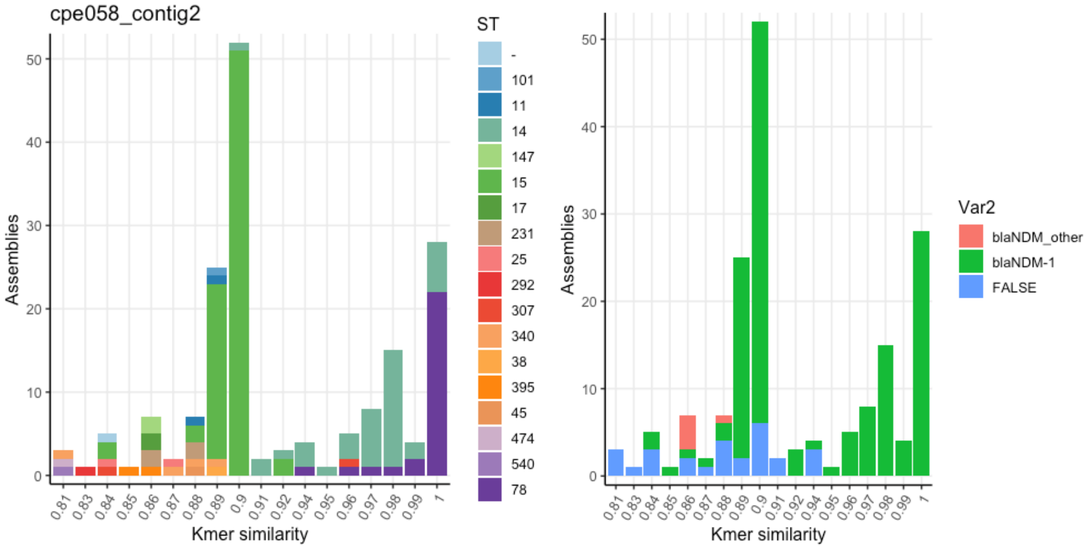

Supplementary Figure 9: isolates found to have >0.8 (80%) kmer identity to the outbreak IncFIB/IncHI1B plasmid (reference cpe058_contig2). Left: Kmer-similarity vs. number of assemblies coloured by Sequence Type (ST) (note, all isolates found here were *Klebsiella pneumoniae*). Right: Kmer-similarity vs. number of assemblies coloured by NDM type. FALSE = no NDM gene found.

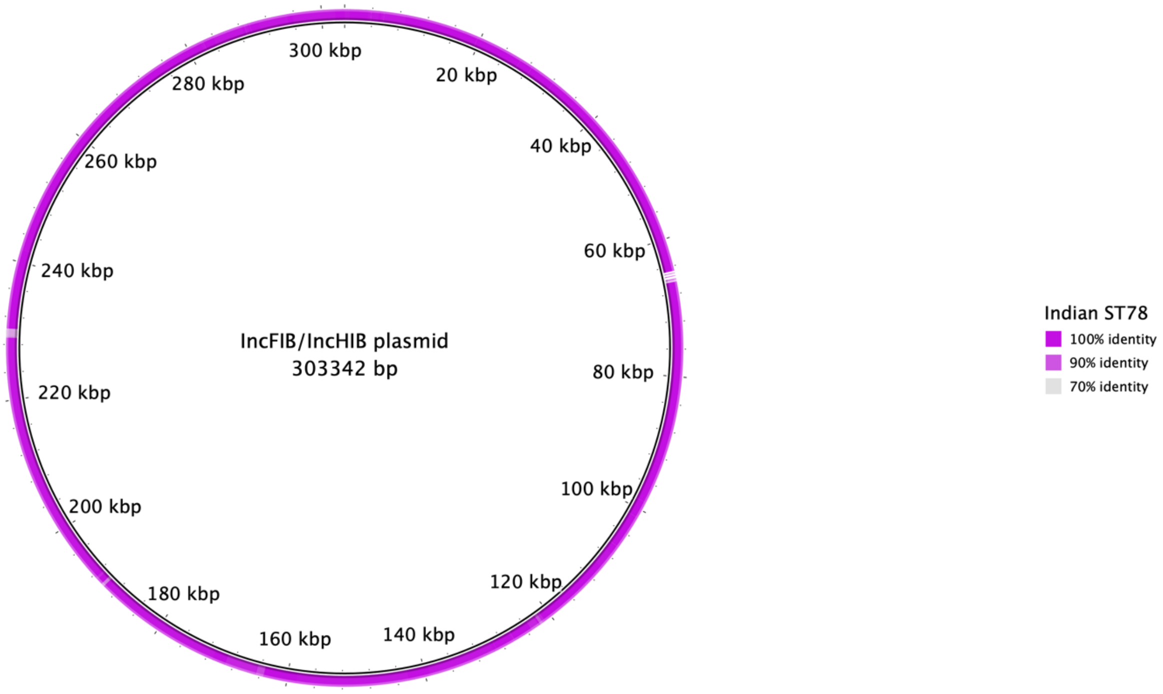

Supplementary Figure 10: BRIG image of Indian ST78 isolate compared to IncFIB/IncHIB NDM-1+ plasmid. Purple ring shows nucleotide blast identity to the reference plasmid (black inner ring).

References

1. PHE. Carbapenemase-producing Enterobacteriaceae toolkit published. 2013 [Available from: https://www.gov.uk/government/news/phe-carbapenemase-producing-enterobacteriaceae-toolkit-published.
2. EUCAST. EUCAST guidelines for detection of resistance mechanisms and specific resistances of clinical and/or epidemiological importance (version 2) 2017 [Available from: https://www.eucast.org/fileadmin/src/media/PDFs/EUCAST_files/Resistance_mechanisms/EUCAST_detection_of_resistance_mechanisms_170711.pdf.
3. De Coster W, D'Hert S, Schultz DT, Cruts M, Van Broeckhoven C. NanoPack: visualizing and processing long-read sequencing data. Bioinformatics. 2018;34(15):2666-9.
4. Wick RR. Filtlong. https://githubcom/rrwick/Filtlong. 2017.
5. Kolmogorov M, Yuan J, Lin Y, Pevzner PA. Assembly of long, error-prone reads using repeat graphs. Nat Biotechnol. 2019;37(5):540-6.
6. Wick RR, Judd LM, Gorrie CL, Holt KE. Unicycler: Resolving bacterial genome assemblies from short and long sequencing reads. PLoS Comput Biol. 2017;13(6):e1005595.
7. Koren S, Walenz BP, Berlin K, Miller JR, Bergman NH, Phillippy AM. Canu: scalable and accurate long-read assembly via adaptive k-mer weighting and repeat separation. Genome Res. 2017;27(5):722-36.
8. Hunt M, Silva ND, Otto TD, Parkhill J, Keane JA, Harris SR. Circlator: automated circularization of genome assemblies using long sequencing reads. Genome Biol. 2015;16:294.
9. Hu J, Fan J, Sun Z, Liu S. NextPolish: a fast and efficient genome polishing tool for long-read assembly. Bioinformatics. 2020;36(7):2253-5.
10. Pierce NT IL, Reiter T et al. . Large-scale sequence comparisons with sourmash. F1000Res. 2019;8(1006).
11. Blackwell GA, Hunt M, Malone KM, Lima L, Horesh G, Alako BTF, et al. Exploring bacterial diversity via a curated and searchable snapshot of archived DNA sequences. PLoS Biol. 2021;19(11):e3001421.
12. Lees JA, Harris SR, Tonkin-Hill G, Gladstone RA, Lo SW, Weiser JN, et al. Fast and flexible bacterial genomic epidemiology with PopPUNK. Genome Res. 2019;29(2):304-16.
13. Simonsen M, Mailund, T., Pedersen, C. N. S. Rapid Neighbour Joining. Proceedings of the 8th Workshop in Algorithms in Bioinformatics (WABI). 2008;LNBI 5251:113-22.
14. Yu G, Smith, D. K., Zhu, H.,Guan, Y., Tsan-Yuk Lam, T. ggtree: an r package for visualization and annotation of phylogenetic trees with their covariates and other associated data. Methods in Ecology and Evolution. 2016;8(1):28-36.
15. Paul M, Narendrakumar L, A RV, Joseph I, Thomas S. Genome sequence of a multidrug-resistant Klebsiella pneumoniae ST78 with high colistin resistance isolated from a patient in India. J Glob Antimicrob Resist. 2019;17:187-8.
16. Huang W, Li L, Myers JR, Marth GT. ART: a next-generation sequencing read simulator. Bioinformatics. 2012;28(4):593-4.
17. Bingmann T. BP, Gauger F., Iqbal Z. COBS: A Compact Bit-Sliced Signature Index. In: Brisaboa N, Puglisi S (eds) String Processing and Information Retrieval SPIRE 2019 Lecture Notes in Computer Science. 2019;11811.

Additional File 2: Sample metadata and Accession numbers

Table 1. Sample metadata

patient ID	duplicate	Sample date	Sample type	Strain ID	WGS species ID	Organism	Mech 2	Mech 2	Meropenem	LOS (days)
P_001		13/11/2014	urine			Klebsiella pneumoniae	OXA-48		R	N/A (GP)
P_002		19/11/2014	urine			Escherichia coli	OXA-48		I	N/A (GP)
P_003		26/11/2014	screen			Klebsiella pneumoniae	KPC		R	30
P_003		26/11/2014	screen			Acinetobacter baumannii	OXA-51		R	30
P_003		26/11/2014	screen			Pseudomonas aeruginosa	VIM		R	30
P_004		25/11/2015	urine			Escherichia coli	OXA-48		I	N/A (GP)
P_005		08/04/2016	urine			Escherichia coli	OXA-48		R	N/A (GP)
P_006		15/06/2016	tissue			Citrobacter freundii	NDM-1		R	93
P_007		20/06/2016	screen			Klebsiella pneumoniae	NDM-1		R	36
P_008	outbreak screening	24/12/2017	sputum	cpe033	Klebsiella pneumoniae	Klebsiella pneumoniae	NDM-1		R	70
P_008	outbreak screening	26/12/2017	blood	cpe034	Klebsiella pneumoniae	Klebsiella pneumoniae	NDM-1		R	70
P_008	outbreak screening	27/12/2017	blood	cpe035	Klebsiella pneumoniae	Klebsiella pneumoniae	NDM-1		R	70
P_008	outbreak screening	15/09/2018	screen	cpe057	Klebsiella pneumoniae	Klebsiella pneumoniae	NDM-1		R	70
P_008	outbreak screening	21/06/2016	blood			Klebsiella pneumoniae	NDM-1		R	70
P_009		27/06/2016	screen			Klebsiella pneumoniae	NDM-1		R	8
P_010		06/07/2016	BAL	cpe001	Klebsiella pneumoniae	Klebsiella pneumoniae	NDM-1		R	73
P_011		09/07/2016	screen			Klebsiella pneumoniae	NDM-1		R	31
P_012		11/07/2016	screen			Klebsiella pneumoniae	NDM-1		R	20
P_013		16/07/2016	screen	cpe002	Enterobacter hormaechei	Enterobacter cloacae	IMP1		I	66
P_014		20/08/2016	screen			Acinetobacter baumannii	OXA-51	OXA-23	R	27
P_015		05/12/2016	screen	cpe003	Klebsiella pneumoniae	Klebsiella pneumoniae	NDM-1		R	26
P_016		09/12/2016	screen	cpe004	Klebsiella pneumoniae	Klebsiella pneumoniae	NDM-1		R	14
P_017		13/12/2016	wound swab	cpe005	Pseudomonas monteilii	Pseudomonas monteilli	IMP1		R	156
P_018	outbreak screening	20/02/2017	screen	cpe059	Klebsiella pneumoniae	Klebsiella pneumoniae	NDM-1		R	48
P_018	outbreak screening	11/05/2017	screen	cpe061	Klebsiella pneumoniae	Klebsiella pneumoniae	NDM-1		R	48
P_018	outbreak screening	13/12/2016	screen			Klebsiella pneumoniae	NDM-1		R	48
P_019	outbreak screening	30/12/2016	urine	cpe058	Klebsiella pneumoniae	Klebsiella pneumoniae	NDM-1		R	37
P_019	outbreak screening	17/12/2016	screen			Klebsiella pneumoniae	NDM-1		R	37
P_020		20/12/2016	tissue	icp157	Enterobacter hormaechei	Enterobacter cloacae	IMP1		R	N/A (no admission)
P_021		10/02/2017	screen	cpe006	Pseudomonas putida	Pseudomonas putida	IMP1		R	10
P_022		17/02/2017	screen	cpe007	Pseudomonas aeruginosa	Pseudomonas aeruginosa	VIM		R	79
P_022		23/02/2017	screen	cpe010	Klebsiella pneumoniae	Klebsiella pneumoniae	NDM-1		R	79
P_022	duplicate	25/08/2017	urine	cpe020	Escherichia coli	Escherichia coli	NDM-1		R	79
P_022	duplicate	22/05/2017	sputum			Escherichia coli	NDM-1		R	79
P_023		18/02/2017	screen	cpe008	Pseudomonas putida	Pseudomonas putida	IMP1		R	17
P_024		18/02/2017	screen			Escherichia coli	OXA-48		I	N/A (no discharge)
P_025		21/02/2017	screen	cpe009	Klebsiella aerogenes	Enterobacter aerogenes	OXA-48		I	13

P_026		08/04/2017	screen	cpe011	Acinetobacter baumannii	Acinetobacter baumannii	OXA-51	OXA-23	R	10
P_027		16/04/2017	screen			Escherichia coli	OXA-48		I	2
P_028		26/04/2017	screen	cpe012	Escherichia coli	Escherichia coli	NDM-1		R	19
P_029		02/06/2017	screen	cpe013	Klebsiella pneumoniae	Klebsiella pneumoniae	NDM-1		R	0
P_029		02/06/2017	screen	cpe014	Escherichia coli	Escherichia coli	NDM-1		R	0
P_030	duplicate	27/07/2017	biliary fluid	cpe015	Citrobacter freundii	Citrobacter freundii	IMP1		I	21
P_030	duplicate	15/08/2017	screen			Citrobacter freundii	IMP1		unknown	21
P_031		14/08/2017	screen	cpe017	Acinetobacter baumannii	Acinetobacter baumannii	OXA-51		I	35
P_032		14/08/2017	screen	cpe016	Acinetobacter baumannii	Acinetobacter baumannii	OXA-51		I	25
P_033		21/08/2017	screen	cpe018	Enterobacter mori	Enterobacter cloacae	IMI		R	35
P_034		24/08/2017	swab	cpe019	Pseudomonas aeruginosa	Pseudomonas aeruginosa	VIM		I	3
P_035		29/09/2017	screen			Escherichia coli	NDM-1		R	5
P_036		14/10/2017	screen	cpe022	Klebsiella pneumoniae	Klebsiella pneumoniae	NDM-1		R	2
P_037		25/10/2017	screen	cpe023	Escherichia coli	Escherichia coli	NDM-1		R	11
P_038		28/10/2017	screen	cpe024	Escherichia coli	Escherichia coli	NDM-1		R	12
P_038		01/11/2017	screen	cpe027	Klebsiella pneumoniae	Klebsiella pneumoniae	OXA-48		R	12
P_039		30/10/2017	urine	cpe025	Citrobacter freundii	Citrobacter freundii	OXA-48		R	N/A (GP)
P_040		01/11/2017	screen	cpe026	Escherichia coli	Escherichia coli	NDM-1		R	51
P_041		06/11/2017	screen	cpe028	Escherichia coli	Escherichia coli	OXA-48		I	26
P_041		06/11/2017	screen	cpe029	Klebsiella pneumoniae	Klebsiella pneumoniae	OXA-48		I	26
P_042		09/11/2017	screen			Pseudomonas putida	VIM		R	14
P_043		23/11/2017	blood			Acinetobacter baumannii	OXA-51	OXA-40	R	79
P_044		17/12/2017	screen	cpe032	Escherichia coli	Escherichia coli	OXA-48		I	7
P_045		28/12/2017	wound swab	cpe037	Acinetobacter pittii	Acinetobacter pittii	OXA-51	NDM-1	R	18
P_046		15/01/2018	screen			Citrobacter freundii	IMP1		R	31
P_047		23/01/2018	screen	cpe040	Escherichia coli	Escherichia coli	OXA-48		I	6
P_048		27/01/2018	screen	cpe041	Escherichia coli	Escherichia coli	OXA-48		I	6
P_049		05/02/2018	screen	cpe042	Enterobacter hormaechei	Enterobacter cloacae	IMP1		I	31
P_050		09/02/2018	urine			Klebsiella pneumoniae	OXA-48		I	N/A (GP)
P_051		17/02/2018	screen	cpe043	Klebsiella pneumoniae	Klebsiella pneumoniae	OXA-48		R	66
P_051		18/02/2018	sputum	cpe044	Acinetobacter baumannii	Acinetobacter baumannii	OXA-51	OXA-23	R	66
P_052		10/03/2018	screen			Acinetobacter baumannii	OXA-51	OXA-40	R	13
P_053		26/03/2018	screen	cpe045	Acinetobacter baumannii	Acinetobacter baumannii	OXA-51	OXA-23	R	72
P_054		06/04/2018	screen	cpe046	Escherichia coli	Escherichia coli	OXA-48		I	15
P_055		25/04/2018	screen			Escherichia coli	OXA-48		I	6
P_055		25/04/2018	screen			Klebsiella pneumoniae	OXA-48		I	6
P_056		04/05/2018	screen	cpe047	Klebsiella pneumoniae	Klebsiella pneumoniae	OXA-48		R	13
P_057		04/05/2018	screen	cpe048	Acinetobacter baumannii	Acinetobacter baumannii	OXA-51	OXA-40	R	39
P_058		11/05/2018	screen			Klebsiella pneumoniae	OXA-48		S	N/A (no admission)
P_059		17/05/2018	sputum	cpe049	Klebsiella pneumoniae	Klebsiella pneumoniae	NDM-1		R	N/A (no admission)
P_060		28/05/2018	screen	cpe050	Enterobacter hormaechei	Enterobacter kobei	OXA-48		R	3
P_061		01/06/2018	blood	cpe055	Acinetobacter baumannii	Acinetobacter baumannii	OXA-51	OXA-40	R	29
P_062		04/07/2018	screen			Acinetobacter baumannii	OXA-51		R	89

P_063		07/07/2018	screen			Escherichia coli	NDM-1		R	3
P_064		21/07/2018	screen	cpe051	Escherichia coli	Escherichia coli	OXA-48		S	5
P_064		21/07/2018	screen	cpe052	Klebsiella pneumoniae	Klebsiella pneumoniae	OXA-48		R	5
P_065		08/08/2018	screen	cpe053	Pseudomonas aeruginosa	Pseudomonas aeruginosa	IMP1		R	35
P_066		17/08/2018	screen			Klebsiella pneumoniae	OXA-48		R	37
P_067		27/08/2018	screen			Klebsiella pneumoniae	OXA-48		R	2
P_068		02/10/2018	urine			Escherichia coli	NDM-1		R	11
P_069		13/10/2018	screen	cpe067	Escherichia coli	Escherichia coli	OXA-48		S	2
P_070		17/10/2018	screen	cpe076	Klebsiella pneumoniae	Klebsiella pneumoniae	KPC		I	45
P_071		22/10/2018	body fluid	cpe079	Pseudomonas aeruginosa	Pseudomonas aeruginosa	VIM		R	58
P_071		30/10/2018	screen			Acinetobacter baumannii	OXA-51	OXA-40	R	58
P_072		25/10/2018	biliary fluid	cpe077	Acinetobacter baumannii	Acinetobacter baumannii	OXA-51	OXA-40	R	75
P_073		26/10/2018	screen	cpe078	Escherichia coli	Escherichia coli	NDM-1		I	58
P_074		12/11/2018	screen	cpe080	Acinetobacter baumannii	Acinetobacter baumannii	OXA-51	OXA-23	R	7
P_075		26/11/2018	screen			Enterobacter cloacae	IMP1		I	25
P_076		19/12/2018	screen			Citrobacter freundii	NDM-1		I	48
P_077		21/12/2018	urine			Klebsiella pneumoniae	NDM-1		R	46
P_078		28/12/2018	screen	cpe081	Escherichia coli	Escherichia coli	NDM-1		R	6
P_079		30/12/2018	screen	cpe082	Escherichia coli	Escherichia coli	NDM-1		I	7
P_080		30/12/2018	screen	cpe083	Klebsiella pneumoniae	Klebsiella pneumoniae	OXA-48		I	27
P_081		03/01/2019	sputum			Pseudomonas aeruginosa	NDM-1		R	N/A (no admission)
P_082		29/01/2019	urine	cpe084	Escherichia coli	Escherichia coli	NDM-1		I	N/A (GP)
P_083		31/01/2019	screen	cpe068	Klebsiella pneumoniae	Klebsiella pneumoniae	OXA-48		S	27
P_084		01/02/2019	screen	cpe069	Escherichia coli	Escherichia coli	NDM-1		R	25
P_084		01/02/2019	screen	cpe070	Klebsiella pneumoniae	Klebsiella pneumoniae	NDM-1		R	25
P_085		05/02/2019	screen			Klebsiella pneumoniae	OXA-48		I	8
P_086		06/02/2019	screen	cpe072	Escherichia coli	Escherichia coli	NDM-1		I	19
P_086		06/02/2019	screen	cpe073	Klebsiella pneumoniae	Klebsiella pneumoniae	NDM-1		R	19
P_087		14/02/2019	CVC tip	cpe085	Klebsiella pneumoniae	Klebsiella pneumoniae	OXA-48		R	9
P_088		19/02/2019	screen	cpe086	Klebsiella pneumoniae	Klebsiella pneumoniae	OXA-48		R	73
P_088		19/02/2019	screen	cpe087	Escherichia coli	Escherichia coli	NDM-1		R	73
P_089		20/02/2019	blood	cpe088	Klebsiella oxytoca	Klebsiella pneumoniae	OXA-48		R	56
P_090		12/03/2019	screen			Pantoea spp.	IMP1		I	5
P_091		14/03/2019	screen	cpe090	Enterobacter hormaechei	Enterobacter cloacae	IMP1		I	2
P_092		16/03/2019	screen			Escherichia coli	NDM-1		R	5
P_093		10/04/2019	screen	cpe091	Escherichia coli	Escherichia coli	OXA-48		S	6
P_094		11/04/2019	screen	cpe092	Escherichia coli	Escherichia coli	OXA-48		S	50
P_095		24/04/2019	urine			Klebsiella pneumoniae	OXA-48		R	N/A (no admission)
P_096		18/05/2019	screen			Klebsiella pneumoniae	OXA-48		S	13
P_097		14/06/2019	screen	cpe093	Enterobacter hormaechei	Enterobacter cloacae	NDM-1		R	2
P_098		20/06/2019	screen			Escherichia coli	OXA-48		S	10
P_099		29/06/2019	screen			Escherichia coli	NDM-1		R	8
P_100		07/07/2019	screen			Escherichia coli	OXA-48		S	4

P_101		22/07/2019	body fluid			Citrobacter freundii	NDM-1		R	5
P_102		23/09/2019	screen			Citrobacter freundii	IMP1		I	79
P_103		01/10/2019	screen	cpe097	Klebsiella quasipneumoniae	Klebsiella pneumoniae	NDM-1		R	N/A (no discharge)
P_104	duplicate	15/10/2019	screen	cpe098	Escherichia coli	Escherichia coli	OXA-48		S	29
P_104	duplicate	18/10/2019	PTC fluid	cpe100	Escherichia coli	Escherichia coli	OXA-48		unknown	29
P_104	duplicate	18/10/2019	PTC fluid	cpe102	Klebsiella grimontii	Klebsiella oxytoca	OXA-48		unknown	29
P_104	duplicate	18/10/2019	PTC fluid	cpe104	Enterobacter roggenkampii	Enterobacter cloacae	OXA-48		unknown	29
P_104		18/10/2019	PTC fluid	cpe105	Raoultella ornithinolytica	Raoultella ornithinolytica	OXA-48		unknown	29
P_104	duplicate	18/10/2019	PTC fluid	cpe106	Enterobacter roggenkampii	Enterobacter cloacae	OXA-48		unknown	29
P_104	duplicate	15/10/2019	screen			Enterobacter cloacae	OXA-48		S	29
P_104	duplicate	15/10/2019	screen			Klebsiella oxytoca	OXA-48		S	29
P_104	duplicate	18/10/2019	PTC fluid			Escherichia coli	OXA-48		unknown	29
P_105		23/10/2019	screen			Escherichia coli	OXA-48		S	21
P_106		28/10/2019	screen	cpe099	Escherichia coli	Escherichia coli	NDM-1		R	N/A (no admission)
P_107		29/10/2019	urine			Escherichia coli	OXA-48		S	N/A (no admission)
P_108		06/11/2019	wound swab			Enterobacter cloacae	NDM-1		I	23
P_109		14/11/2019	screen			Escherichia coli	OXA-48		I	7
P_110		19/12/2019	screen			Enterobacter cloacae	IMP1		I	12
P_111		17/12/2019	urine			Escherichia coli	NDM-1		R	N/A (GP)
P_112		21/12/2019	screen			Escherichia coli	OXA-48		I	4
P_113		04/01/2020	screen			Klebsiella oxytoca	KPC		I	4
P_114		02/01/2020	urine			Escherichia coli	OXA-48		I	N/A (GP)
P_115		29/02/2020	screen			Escherichia coli	NDM-1		I	12
P_116		07/03/2020	screen			Escherichia coli	NDM-1		R	N/A (no discharge)
P_117		09/04/2020	screen			Klebsiella pneumoniae	OXA-48		S	no information
P_118		16/04/2020	urine			Escherichia coli	NDM-1		R	29
P_119		19/05/2020	drain			Escherichia coli	NDM-1		R	32
P_120		22/05/2020	screen			Klebsiella pneumoniae	OXA-48		R	4
P_121		22/06/2020	wound swab			Pseudomonas aeruginosa	DIM		R	N/A (no discharge)
P_121		26/06/2020	screen			Escherichia coli	NDM-1		R	N/A (no discharge)
P_122		29/07/2020	screen			Klebsiella pneumoniae	OXA-48		R	39
P_123		17/09/2020	blood			Klebsiella pneumoniae	NDM-1		R	37
P_124		15/09/2020	screen			Escherichia coli	NDM-1		R	1
P_125		08/10/2020	screen			Escherichia coli	NDM-1		R	18
P_126		22/10/2020	urine			Citrobacter koseri	OXA-48		S	N/A (no admission)
P_127		22/10/2020	screen			Pseudomonas putida	VIM		R	1
P_128		28/10/2020	blood			Escherichia coli	NDM-1		R	10
P_129		29/10/2020	screen			Escherichia coli	OXA-48		I	N/A (no discharge)
P_130		02/12/2020	screen			Klebsiella pneumoniae	OXA-48	NDM-1	R	10
P_131		06/12/2020	screen			Klebsiella pneumoniae	NDM-1		R	no information
P_132		28/12/2020	blood			Klebsiella pneumoniae	NDM-1		R	no information
P_133		29/03/2017	screen	cpe060	Klebsiella pneumoniae	Klebsiella pneumoniae	OXA-48		unknown	N/A (no discharge)

Table 2: Sample accession numbers

strain	study_accession	sample_accession	Illumina accession	Illumina sample title	nanopore barcode	ONT reads	ONT read accessio	Illumina lane	WGS species	collection_date	country	host	isolation_source	serovar	carbapenema	Assembler	assembly size (bp	number of contigs	assembly quality
cpe001	PRJEB30134	SAMEA5226442	ERR4095905	BRCWGS7756684	cpe02_3.6.0_bc01	cpe001.fastq.gz	ERR8282738	29210_5#32	Klebsiella pneumoniae	2016-07-06	United Kingdom	human	bronchoalveolar lava	not known	NDM-1	canu (circlator)	6027395	5	incomplete (chr present)
cpe002	PRJEB30134	SAMEA6451145	ERR5386282	BRCWGS8710437	cpe02_3.6.0_bc02	cpe002.fastq.gz	ERR8282739	33431_1#6	Enterobacter hormaechei	2016-07-16	United Kingdom	human	screen	not known	IMP-1/IMP-70	canu (circlator)	5068852	6	incomplete (chr present)
cpe003	PRJEB30134	SAMEA5226443	ERR4095908	BRCWGS7756685	cpe02_3.6.0_bc03	cpe003.fastq.gz	ERR8282740	29210_5#35	Klebsiella pneumoniae	2016-12-05	United Kingdom	human	screen	not known	NDM-1	flye	5794252	4	complete
cpe004	PRJEB30134	SAMEA5226444	ERR4095909	BRCWGS7756686	cpe02_3.6.0_bc04	cpe004.fastq.gz	ERR8282741	29210_5#36	Klebsiella pneumoniae	2016-12-09	United Kingdom	human	screen	not known	NDM-1	unicycler	5761699	4	complete
cpe005	PRJEB30134	SAMEA5226445	ERR4095912	BRCWGS7756687	cpe02_3.6.0_bc05	cpe005.fastq.gz	ERR8282742	29210_5#39	Pseudomonas monteilii	2016-12-13	United Kingdom	human	wound swab	not known	IMP-15	flye	6503588	2	complete
cpe006	PRJEB30134	SAMEA5226446	ERR4095913	BRCWGS7756688	cpe02_3.6.0_bc06	cpe006.fastq.gz	ERR8282743	29210_5#40	Pseudomonas putida	2017-02-10	United Kingdom	human	screen	not known	IMP-1/IMP-70	flye	6790848	2	complete
cpe007	PRJEB30134	SAMEA5226447	ERR4095915	BRCWGS7756689	cpe02_3.6.0_bc07	cpe007.fastq.gz	ERR8282744	29210_5#42	Pseudomonas aeruginosa	2017-02-17	United Kingdom	human	screen	not known	VIM-5	flye	6883014	3	complete
cpe008	PRJEB30134	SAMEA5226448	ERR4095918	BRCWGS7756690	cpe02_3.6.0_bc08	cpe008.fastq.gz	ERR8282745	29210_5#45	Pseudomonas putida	2017-02-18	United Kingdom	human	screen	not known	IMP-1/IMP-70	unicycler	6785872	3	complete
cpe009	PRJEB30134	SAMEA6451149	ERR5386284	BRCWGS8710438	cpe02_3.6.0_bc09	cpe009.fastq.gz	ERR8282746	33431_1#8	Klebsiella aerogenes	2017-02-21	United Kingdom	human	screen	not known	OXA-48	flye (filtered)	5425747	3	complete
cpe010	PRJEB30134	SAMEA6451150	ERR5386286	BRCWGS8710439	cpe02_3.6.0_bc10	cpe010.fastq.gz	ERR8282747	33431_1#10	Klebsiella pneumoniae	2017-02-23	United Kingdom	human	screen	not known	NDM-1	unicycler	5970701	9	complete
cpe011	PRJEB30134	SAMEA6451152	ERR5386288	BRCWGS8710440	cpe03_3.6.0_bc01	cpe011.fastq.gz	ERR8282748	33431_1#12	Acinetobacter baumannii	2017-04-08	United Kingdom	human	screen	not known	OXA-23/OXA-66	unicycler	4127447	2	complete
cpe012	PRJEB30134	SAMEA6451147	ERR5386290	BRCWGS8710441	cpe01_3.6.0_bc01	cpe012.fastq.gz	ERR8282749	33431_1#14	Escherichia coli	2017-04-26	United Kingdom	human	screen	not known	NDM-1	flye	5535697	6	complete
cpe013	PRJEB30134	SAMEA5226449	ERR4095919	BRCWGS7756691	cpe03_3.6.0_bc02	cpe013.fastq.gz	ERR8282750	29210_5#46	Klebsiella pneumoniae	2017-06-02	United Kingdom	human	screen	not known	NDM-6	unicycler	5670311	5	complete
cpe014	PRJEB30134	SAMEA5226450	ERR4095922	BRCWGS7756692	cpe03_3.6.0_bc03	cpe014.fastq.gz	ERR8282751	29210_5#49	Escherichia coli	2017-06-02	United Kingdom	human	screen	not known	NDM-6	flye	5030247	3	complete
cpe015	PRJEB30134	SAMEA6451148	ERR5386291	BRCWGS8710442	cpe03_3.6.0_bc04	cpe015.fastq.gz	ERR8282752	33431_1#15	Citrobacter freundii	2017-07-27	United Kingdom	human	biliary fluid	not known	IMP-1/IMP-70	flye	5292210	4	complete
cpe016	PRJEB30134	SAMEA5226451	ERR4095907	BRCWGS7756693	cpe03_3.6.0_bc05	cpe016.fastq.gz	ERR8282753	29210_5#34	Acinetobacter baumannii	2017-08-14	United Kingdom	human	screen	not known	OXA-78	flye	3971187	5	complete
cpe017	PRJEB30134	SAMEA6451151	ERR5386293	BRCWGS8710443	cpe03_3.6.0_bc06	cpe017.fastq.gz	ERR8282754	33431_1#17	Acinetobacter baumannii	2017-08-14	United Kingdom	human	screen	not known	OXA-64	unicycler	3926402	2	complete
cpe018	PRJEB30134	SAMEA6451153	ERR5386295	BRCWGS8710444	cpe03_3.6.0_bc07	cpe018.fastq.gz	ERR8282755	33431_1#19	Enterobacter mori	2017-08-21	United Kingdom	human	screen	not known	IMI-2	flye	5338680	4	complete
cpe019	PRJEB30134	SAMEA5226452	ERR4095923	BRCWGS7756694	cpe03_3.6.0_bc08	cpe019.fastq.gz	ERR8282756	29210_5#50	Pseudomonas aeruginosa	2017-08-24	United Kingdom	human	swab	not known	VIM-47	unicycler	6917874	1	complete
cpe020	PRJEB30134	SAMEA6451156	ERR5386297	BRCWGS8710445	cpe03_3.6.0_bc09	cpe020.fastq.gz	ERR8282757	33431_1#21	Escherichia coli	2017-08-25	United Kingdom	human	urine	not known	NIL: remove	unicycler	5349997	5	complete
cpe022	PRJEB30134	SAMEA6451160	ERR5386299	BRCWGS8710446	cpe06_3.6.0_bc01	cpe022.fastq.gz	ERR8282758	33431_1#23	Klebsiella pneumoniae	2017-10-14	United Kingdom	human	screen	not known	NDM-1	flye	5749328	6	complete
cpe023	PRJEB30134	SAMEA5226454	ERR4095927	BRCWGS7756696	cpe01_3.6.0_bc02	cpe023.fastq.gz	ERR8282759	29210_5#54	Escherichia coli	2017-10-25	United Kingdom	human	screen	not known	NDM-5	flye	5332485	4	complete
cpe024	PRJEB30134	SAMEA6451154	ERR5386301	BRCWGS8710447	cpe06_3.6.0_bc02	cpe024.fastq.gz	ERR8282760	33431_1#25	Escherichia coli	2017-10-28	United Kingdom	human	screen	not known	NDM-5	flye	5548216	6	complete
cpe025	PRJEB30134	SAMEA6451155	ERR5386303	BRCWGS8710448	cpe06_3.6.0_bc03	cpe025.fastq.gz	ERR8282761	33431_1#27	Citrobacter freundii	2017-10-30	United Kingdom	human	urine	not known	OXA-48	flye	5450448	7	complete
cpe026	PRJEB30134	SAMEA5226455	ERR4095930	BRCWGS7756697	cpe01_3.6.0_bc03	cpe026.fastq.gz	ERR8282762	29210_5#57	Escherichia coli	2017-11-01	United Kingdom	human	screen	not known	NDM-5	unicycler (circlator)	5499758	6	incomplete (chr present)
cpe027	PRJEB30134	SAMEA6451157	ERR5386306	BRCWGS8710449	cpe06_3.6.0_bc04	cpe027.fastq.gz	ERR8282763	33431_1#30	Klebsiella pneumoniae	2017-11-01	United Kingdom	human	screen	not known	OXA-232	unicycler	5657996	7	complete
cpe028	PRJEB30134	SAMEA6451159	ERR5386308	BRCWGS8710450	cpe06_3.6.0_bc05	cpe028.fastq.gz	ERR8282764	33431_1#32	Escherichia coli	2017-11-06	United Kingdom	human	screen	not known	OXA-48	flye (filtered)	5138903	4	complete
cpe029	PRJEB30134	SAMEA6451158	ERR5386310	BRCWGS8710451	cpe06_3.6.0_bc06	cpe029.fastq.gz	ERR8282765	33431_1#34	Klebsiella pneumoniae	2017-11-06	United Kingdom	human	screen	not known	OXA-48	flye (filtered)	5736398	8	complete
cpe032	PRJEB30134	SAMEA6451166	ERR5386312	BRCWGS8710452	cpe06_3.6.0_bc08	cpe032.fastq.gz	ERR8282766	33431_1#36	Escherichia coli	2017-12-17	United Kingdom	human	screen	not known	OXA-48	flye	5246553	1	complete
cpe033	PRJEB30134	SAMEA5226458	ERR4095935	BRCWGS7756700	cpe06_3.6.0_bc09	cpe033.fastq.gz	ERR8282767	29210_5#62	Klebsiella pneumoniae	2017-12-24	United Kingdom	human	sputum	not known	NDM-1	unicycler (circlator)	5937423	5	incomplete (chr present)
cpe034	PRJEB30134	SAMEA5226459	ERR4095910	BRCWGS7756701	cpe06_3.6.0_bc10	cpe034.fastq.gz	ERR8282768	29210_5#37	Klebsiella pneumoniae	2017-12-26	United Kingdom	human	blood	not known	NDM-1	unicycler	5935972	5	complete
cpe035	PRJEB30134	SAMEA5226460	ERR4095938	BRCWGS7756702	cpe04_3.6.0_bc01	cpe035.fastq.gz	ERR8282769	29210_5#65	Klebsiella pneumoniae	2017-12-27	United Kingdom	human	blood	not known	NDM-1	flye	5940609	5	complete
cpe037	PRJEB30134	SAMEA5226462	ERR4095939	BRCWGS7756704	cpe04_3.6.0_bc03	cpe037.fastq.gz	ERR8282770	29210_5#66	Acinetobacter pittii	2017-12-28	United Kingdom	human	wound swab	not known	OXA-421	flye	4463512	6	complete
cpe040	PRJEB30134	SAMEA5226465	ERR4095942	BRCWGS7756707	cpe04_3.6.0_bc06	cpe040.fastq.gz	ERR8282771	29210_5#69	Escherichia coli	2018-01-23	United Kingdom	human	screen	not known	OXA-48	flye (filtered)	5537160	8	complete
cpe041	PRJEB30134	SAMEA5226466	ERR4095943	BRCWGS7756708	cpe04_3.6.0_bc07	cpe041.fastq.gz	ERR8282772	29210_5#70	Escherichia coli	2018-01-27	United Kingdom	human	screen	not known	OXA-181	flye	5043730	5	complete
cpe042	PRJEB30134	SAMEA5226467	ERR4095946	BRCWGS7756709	cpe04_3.6.0_bc08	cpe042.fastq.gz	ERR8282773	29210_5#73	Enterobacter hormaechei	2018-02-05	United Kingdom	human	screen	not known	IMP-1/IMP-70	unicycler	4970658	5	complete
cpe043	PRJEB30134	SAMEA5226468	ERR4095947	BRCWGS7756710	cpe04_3.6.0_bc09	cpe043.fastq.gz	ERR8282774	29210_5#74	Klebsiella pneumoniae	2018-02-17	United Kingdom	human	screen	not known	OXA-48	unicycler	5656193	6	complete
cpe044	PRJEB30134	SAMEA5226469	ERR4095949	BRCWGS7756711	cpe04_3.6.0_bc10	cpe044.fastq.gz	ERR8282775	29210_5#76	Acinetobacter baumannii	2018-02-18	United Kingdom	human	sputum	not known	OXA-23/OXA-66	flye	4352890	4	complete
cpe045	PRJEB30134	SAMEA5226470	ERR4095952	BRCWGS7756712	cpe05_3.6.0_bc01	cpe045.fastq.gz	ERR8282776	29210_5#79	Acinetobacter baumannii	2018-03-26	United Kingdom	human	screen	not known	OXA-23/OXA-66	unicycler	3990065	3	complete
cpe046	PRJEB30134	SAMEA5226471	ERR4095953	BRCWGS7756713	cpe05_3.6.0_bc02	cpe046.fastq.gz	ERR8282777	29210_5#80	Escherichia coli	2018-04-06	United Kingdom	human	screen	not known	OXA-181	canu (circlator)	5152119	6	incomplete (chr present)
cpe047	PRJEB30134	SAMEA5226472	ERR4095956	BRCWGS7756714	cpe05_3.6.0_bc03	cpe047.fastq.gz	ERR8282778	29210_5#83	Klebsiella pneumoniae	2018-05-04	United Kingdom	human	screen	not known	OXA-232	unicycler	5748054	11	complete
cpe048	PRJEB30134	SAMEA5226473	ERR4095957	BRCWGS7756715	cpe05_3.6.0_bc04	cpe048.fastq.gz	ERR8282779	29210_5#84	Acinetobacter baumannii	2018-05-04	United Kingdom	human	screen	not known	OXA-72/OXA-91	unicycler	3733822	2	complete
cpe049	PRJEB30134	SAMEA5226474	ERR4095960	BRCWGS7756716	cpe05_3.6.0_bc05	cpe049.fastq.gz	ERR8282780	29210_5#87	Klebsiella pneumoniae	2018-05-17	United Kingdom	human	sputum	not known	NDM-5	flye	5552644	5	complete
cpe050	PRJEB30134	SAMEA5226475	ERR4095961	BRCWGS7756717	cpe05_3.6.0_bc06	cpe050.fastq.gz	ERR8282781	29210_5#88	Enterobacter hormaechei	2018-05-28	United Kingdom	human	screen	not known	OXA-181	flye (circlator)	5268464	4	incomplete (chr present)
cpe051	PRJEB30134	SAMEA5226476	ERR4095964	BRCWGS7756718	cpe05_3.6.0_bc07	cpe051.fastq.gz	ERR8282782	29210_5#91	Escherichia coli	2018-07-21	United Kingdom	human	screen	not known	OXA-181	flye	4911074	4	complete
cpe052	PRJEB30134	SAMEA5226477	ERR4095965	BRCWGS7756719	cpe05_3.6.0_bc08	cpe052.fastq.gz	ERR8282783	29210_5#92	Klebsiella pneumoniae	2018-07-21	United Kingdom	human	screen	not known	OXA-232	unicycler	5552711	6	complete
cpe053	PRJEB30134	SAMEA5226478	ERR4095968	BRCWGS7756720	cpe05_3.6.0_bc09	cpe053.fastq.gz	ERR8282784	29210_5#95	Pseudomonas aeruginosa	2018-08-08	United Kingdom	human	screen	not known	IMP-1	unicycler (circlator)	7110879	2	incomplete (chr not present/>15 contigs)
cpe055	PRJEB30134	SAMEA5226480	ERR4095972	BRCWGS7756722	cpe08_3.6.0_bc01	cpe055.fastq.gz	ERR8282785	29210_5#99	Acinetobacter baumannii	2018-06-01	United Kingdom	human	blood	not known	OXA-72/OXA-66	unicycler	3917331	2	complete
cpe057	PRJEB30134	SAMEA5226482	ERR4095976	BRCWGS7756724	cpe08_3.6.0_bc03	cpe057.fastq.gz	ERR8282786	29210_5#103	Klebsiella pneumoniae	2016-12-30	United Kingdom	human	screen	not known	NDM-1	unicycler (circlator)	5789025	6	incomplete (chr present)
cpe058	PRJEB30134	SAMEA5226483	ERR4095977	BRCWGS7756725	cpe08_3.6.0_bc04	cpe058.fastq.gz	ERR8282787	29210_5#104	Klebsiella pneumoniae	2017-02-20	United Kingdom	human	urine	not known	NDM-1	flye	5791376	4	complete
cpe059	PRJEB30134	SAMEA5226484	ERR4095917	BRCWGS7756726	cpe08_3.6.0_bc05	cpe059.fastq.gz	ERR8282788	29210_5#44	Klebsiella pneumoniae	2017-03-29	United Kingdom	human	screen	not known	NDM-1	flye	5793223	4	complete
cpe060	PRJEB30134	SAMEA5226485	ERR4095979	BRCWGS7756727	cpe08_3.6.0_bc06	cpe060.fastq.gz	ERR8282789	29210_5#106	Klebsiella pneumoniae	2017-05-11	United Kingdom	human	screen	not known	OXA-232	unicycler	5569369	8	complete
cpe061	PRJEB30134	SAMEA5226486	ERR4095982	BRCWGS7756728	cpe08_3.6.0_bc07	cpe061.fastq.gz	ERR8282790	29210_5#109	Klebsiella pneumoniae	2018-10-13	United Kingdom	human	screen	not known	NDM-1	flye (filtered)	5807241	3	complete
cpe067	PRJEB30134	SAMEA6451161	ERR5386316	BRCWGS8710454	cpe09_3.6.0_bc02	cpe067.fastq.gz	ERR8282791	33431_1#40	Escherichia coli	2018-10-13	United Kingdom	human	screen	not known	OXA-181	unicycler	5253396	8	complete
cpe068	PRJEB30134	SAMEA6451164	ERR5386318	BRCWGS8710455	cpe09_3.6.0_bc03	cpe068.fastq.gz	ERR8282792	33431_1#42	Klebsiella pneumoniae	2019-01-31	United Kingdom	human	screen	not known	OXA-48	flye	5616738	4	complete
cpe069	PRJEB30134	SAMEA6451163	ERR5386320	BRCWGS8710456	cpe09_3.6.0_bc04	cpe069.fastq.gz	ERR8282793	33431_1#44	Escherichia coli	2019-02-01	United Kingdom	human	screen	not known	NDM-1	flye	4846961	2	complete
cpe070	PRJEB30134	SAMEA6451165	ERR5386322	BRCWGS8710457	cpe09_3.6.0_bc05	cpe070.fastq.gz	ERR8282794	33431_1#46	Klebsiella pneumoniae	2019-02-01	United Kingdom	human	screen	not known	NDM-1	unicycler	5855722	7	complete
cpe072	PRJEB30134	SAMEA6451169	ERR5386326	BRCWGS8710459	cpe09_3.6.0_bc07	cpe072.fastq.gz	ERR8282795	33431_1#50	Escherichia coli	2019-02-06	United Kingdom	human	screen	not known	NDM-1	unicycler	5408306	6	complete
cpe073	PRJEB30134	SAMEA6451170	ERR5386328	BRCWGS8710460	cpe09_3.6.0_bc08	cpe073.fastq.gz	ERR8282796	33431_1#52	Klebsiella pneumoniae	2019-02-06	United Kingdom	human	screen	not known	NDM-1	unicycler	5880992	10	complete
cpe076	PRJEB30134	SAMEA6451094	ERR5386374	BRCWGS8710384	cpe10_3.6.0_bc07	cpe076.fastq.gz	ERR8282797	33431_1#98	Klebsiella pneumoniae	2018-10-17	United Kingdom	human	screen	not known	KPC-3	unicycler	5756715	4	complete
cpe077	PRJEB30134	SAMEA6451095	ERR5386376	BRCWGS8710385	cpe10_3.6.0_bc08	cpe077.fastq.gz	ERR8282798	33431_1#100	Acinetobacter baumannii	2018-10-25	United Kingdom	human	biliary fluid	not known	OXA-72/OXA-66	unicycler	4056697	2	complete
cpe078	PRJEB30134	SAMEA6451093	ERR5386378	BRCWGS8710386	cpe11_3.6.0_bc01	cpe078.fastq.gz	ERR8282799	33431_1#102	Escherichia coli	2018-10-26	United Kingdom	human	screen	not known	NIL: remove	flye	5219785	4	complete
cpe079	PRJEB30134	SAMEA6451098	ERR5386380	BRCWGS8710387	cpe11_3.6.0_bc02	cpe079.fastq.gz	ERR8282800	33431_1#104	Pseudomonas aeruginosa	2018-10-30	United Kingdom	human	body fluid	not known	VIM-36	flye	7104768	3	complete
cpe080	PRJEB30134	SAMEA6451101	ERR5386382	BRCWGS8710388	cpe11_3.6.0_bc03	cpe080.fastq.gz	ERR8282801	33431_1#106	Acinetobacter baumannii	2018-11-12	United Kingdom	human	screen	not known	OXA-23/OXA-66	flye	3957777	4	complete
cpe081	PRJEB30134	SAMEA6451097	ERR5386384	BRCWGS8710389	cpe12_3.6.0_bc04	cpe081.fastq.gz	ERR8282802	33431_1#108	Escherichia coli	2018-12-28	United Kingdom	human	screen	not known	NDM-5	unicycler	5188728	4	complete
cpe082	PRJEB30134	SAMEA6451096	ERR5386386	BRCWGS8710390	cpe12_3.6.0_bc05	cpe082.fastq.gz	ERR8282803	33431_1#110	Escherichia coli	2018-12-30	United Kingdom	human	screen	not known	NDM-5	unicycler	4763927	4	complete
cpe083	PRJEB30134	SAMEA6451099	ERR5386387	BRCWGS8710391	cpe12_3.6.0_bc06	cpe083.fastq.gz	ERR8282804	33431_1#111	Klebsiella pneumoniae	2018-12-30	United Kingdom	human	screen	not known	OXA-181	unicycler	5636513	5	complete
cpe084	PRJEB30134	SAMEA6451100	ERR5386389	BRCWGS8710392	cpe13_3.6.0_bc07	cpe084.fastq.gz	ERR8282805	33431_1#113	Escherichia coli	2019-01-29	United Kingdom	human	urine	not known	NDM-5	flye	5594469	5	complete
cpe085	PRJEB30134	SAMEA6451102	ERR5386391	BRCWGS8710393	cpe13_3.6.0_bc08	cpe085.fastq.gz	ERR8282806	33431_1#115	Klebsiella pneumoniae	2019-02-14	United Kingdom	human	central venous cathe	not known	OXA-232	unicycler	5651001	6	complete
cpe086	PRJEB30134	SAMEA6451103	ERR5386393	BRCWGS8710394	cpe13_3.6.0_bc09	cpe086.fastq.gz	ERR8282807	33431_1#117	Klebsiella pneumoniae	2019-02-19	United Kingdom	human	screen	not known	OXA-232	flye	5613302	6	complete
cpe087	PRJEB30134	SAMEA6451104	ERR5386395	BRCWGS8710395	cpe14_3.6.0_bc01	cpe087.fastq.gz	ERR8282808	33431_1#119	Escherichia coli	2019-02-19	United Kingdom	human	screen	not known	NDM-5	flye	5435324	4	complete
cpe088	PRJEB30134	SAMEA6451105	ERR5386397	BRCWGS8710396	cpe14_3.6.0_bc02	cpe088.fastq.gz	ERR8282809	33431_1#121	Klebsiella oxytoca	2019-02-20	United Kingdom	human	blood	not known	NIL: remove	flye	6304358	4	complete
cpe090	PRJEB30134	SAMEA6451110	ERR5386401	BRCWGS8710398	cpe14_3.6.0_bc04	cpe090.fastq.gz	ERR8282810	33431_1#125	Enterobacter hormaechei	2019-03-14	United Kingdom	human	screen	not known	IMP-1/IMP-70	flye	5020081	6	complete
cpe091	PRJEB30134	SAMEA6451106	ERR5386403	BRCWGS8710399	cpe14_3.6.0_bc05	cpe091.fastq.gz	ERR8282811	33431_1#127	Escherichia coli	2019-04-10	United Kingdom	human	screen	not known	OXA-232	unicycler (circlator)	5069522	12	incomplete (chr not present/>15 contigs)
cpe092	PRJEB30134	SAMEA6451108	ERR5386405	BRCWGS8710400	cpe14_3.6.0_bc06	cpe092.fastq.gz	ERR8282812	33431_1#129	Escherichia coli	2019-04-11	United Kingdom	human	screen	not known	OXA-232	flye	5079763	8	complete
cpe093	PRJEB30134	SAMEA6451109	ERR5386407	BRCWGS8710401	cpe14_3.6.0_bc07	cpe093.fastq.gz	ERR8282813	33431_1#131	Enterobacter hormaechei	2019-06-14	United Kingdom	human	screen	not known	NDM-1	unicycler	5230177	6	complete
cpe097	PRJEB30134	SAMEA6451113	ERR5386416	BRCWGS8710405	cpe14_3.6.0_bc11	cpe097.fastq.gz	ERR8282814	33431_1#140	Klebsiella quasipneumoniae subsp	2019-10-01	United Kingdom	human	screen	not known	NDM-1	flye	5478993	3	complete
cpe098	PRJEB30134	SAMEA6451116	ERR5430236	BRCWGS8710406	cpe14_3.6.0_bc12	cpe098.fastq.gz	ERR8282815	33515_3#167	Escherichia coli	2019-10-15	United Kingdom	human	screen	not known	OXA-181	flye	4890240	7	complete
cpe099	PRJEB30134	SAMEA6451114	ERR5430239	BRCWGS8710407	cpe15_3.6.0_bc01	cpe099.fastq.gz	ERR8282816	33515_3#170	Escherichia coli	2019-10-28	United Kingdom	human	screen	not known	NDM-5	unicycler	5108814	5	complete
cpe100	PRJEB30134	SAMEA6451115	ERR5386418	BRCWGS8710408	cpe15_3.6.0_bc02	cpe100.fastq.gz	ERR8282817	33431_1#142	Escherichia coli	2019-10-18	United Kingdom	human	PTC fluid	not known	NIL: remove	canu (circlator)	4800838	9	incomplete (chr present)
cpe102	PRJEB30134	SAMEA6451123	ERR5386422	BRCWGS8710410	cpe15_3.6.0_bc04	cpe102.fastq.gz	ERR8282818	33431_1#146	Klebsiella grimontii	2019-10-18	United Kingdom	human	PTC fluid	not known	OXA-181	flye	6213454	6	complete
cpe104	PRJEB30134	SAMEA6451119	ERR5386426	BRCWGS8710412	cpe15_3.6.0_bc06	cpe104.fastq.gz	ERR8282819	33431_1#150	Enterobacter roggenkampii	2019-10-18	United Kingdom	human	PTC fluid	not known	OXA-181	unicycler	4858817	6	complete
cpe105	PRJEB30134	SAMEA6451122	ERR5386428	BRCWGS8710413	cpe15_3.6.0_bc07	cpe105.fastq.gz	ERR8282820	33431_1#152	Raoultella ornithinolytica	2019-10-18	United Kingdom	human	PTC fluid	not known	OXA-181	flye	6007147	8	complete
cpe106	PRJEB30134	SAMEA6451121	ERR5386429	BRCWGS8710414	cpe15_3.6.0_bc08	cpe106.fastq.gz	ERR8282821	33431_1#153	Enterobacter roggenkampii	2019-10-18	United Kingdom	human	PTC fluid	not known	OXA-181	flye (circlator)	4991961	7	incomplete (chr present)
icp157	PRJEB30134	SAMEA6451146	ERR5386280	BRCWGS8710436	cpe07_3.6.0_bc03	icp157.fastq.gz	ERR8282822	33431_1#4	Enterobacter hormaechei	2016-12-20	United Kingdom	human	tissue	not known	IMP-1/IMP-70	unicycler	5000369	8	complete
